# TrpM, a Small Protein Modulating Tryptophan Biosynthesis and Morpho-Physiological Differentiation in *Streptomyces coelicolor* A3(2)

**DOI:** 10.1371/journal.pone.0163422

**Published:** 2016-09-26

**Authors:** Emilia Palazzotto, Giuseppe Gallo, Giovanni Renzone, Anna Giardina, Alberto Sutera, Joohee Silva, Celinè Vocat, Luigi Botta, Andrea Scaloni, Anna Maria Puglia

**Affiliations:** 1 Laboratory of Genetics, School of Biosciences and Veterinary Medicine, University of Camerino, 62032 Camerino, Italy; 2 Laboratory of Molecular Microbiology and Biotechnology, STEBICEF Department, University of Palermo, 90128 Palermo, Italy; 3 Proteomic and Mass Spectrometry Laboratory, ISPAAM, National Research Council, 80147 Naples, Italy; 4 Dipartimento di Ingegneria Civile, Ambientale, Aerospaziale, dei Materiali, University of Palermo, 90128 Palermo, Italy; Martin-Luther-Universitat Halle-Wittenberg, GERMANY

## Abstract

In the model actinomycete *Streptomyces coelicolor* A3(2), small open reading frames encoding proteins with unknown functions were identified in several amino acid biosynthetic gene operons, such as *SCO2038* (*trpX*) in the tryptophan *trpCXBA* locus. In this study, the role of the corresponding protein in tryptophan biosynthesis was investigated by combining phenotypic and molecular analyses. The 2038KO mutant strain was characterized by delayed growth, smaller aerial hyphae and reduced production of spores and actinorhodin antibiotic, with respect to the WT strain. The capability of this mutant to grow on minimal medium was rescued by tryptophan and tryptophan precursor (serine and/or indole) supplementation on minimal medium and by gene complementation, revealing the essential role of this protein, here named TrpM, as modulator of tryptophan biosynthesis. His-tag pull-down and bacterial adenylate cyclase-based two hybrid assays revealed TrpM interaction with a putative leucyl-aminopeptidase (PepA), highly conserved component among various *Streptomyces* spp. In *silico* analyses showed that PepA is involved in the metabolism of serine, glycine and cysteine through a network including GlyA, CysK and CysM enzymes. Proteomic experiments suggested a TrpM-dependent regulation of metabolic pathways and cellular processes that includes enzymes such as GlyA, which is required for the biosynthesis of tryptophan precursors and key proteins participating in the morpho-physiological differentiation program. Altogether, these findings reveal that TrpM controls tryptophan biosynthesis at the level of direct precursor availability and, therefore, it is able to exert a crucial effect on the morpho-physiological differentiation program in *S*. *coelicolor* A3(2).

## Introduction

One of the most challenging tasks in the genome sequence era is the assignment of a role to the multitude of predicted proteins having unknown function. Among these are the many intriguing small open reading frames (smORFs) that encode peptides with a length of less than 100 amino acids [1]. To date, many smORFs have been shown to be involved in various cellular processes, such as the regulation of gene expression and of metabolic pathways, and to act as transcriptional regulators, chaperonins, nucleases, or ribosomal, stress response, and membrane proteins [1]. Nevertheless, most of them have to be still characterized.

In the filamentous actinomycete *Streptomyces coelicolor* A3(2), a model organism in the study of bacterial morphological and physiological differentiation, bioinformatic analysis of the genome sequence offers the possibility to identify putative smORFs coding for proteins of unknown function (UF), usually with molecular mass lower than 10 kDa. Interestingly, UF smORFs are often found in the vicinity of ORFs coding for proteins involved in secondary metabolism [2] and biosynthesis of amino acids. A particularly interesting example is the UF *SCO2038* smORF (also called *trpX* [3]) that maps to the *trpCXBA* locus encoding indoleglycerol phosphate synthase (TrpC), tryptophan synthase beta (TrpB) and alpha (TrpA), which are devoted to the last steps of tryptophan (Trp) biosynthesis [3].

In Gram-positive and -negative bacteria, the intracellular level of free Trp and the availability of charged tRNATrp are regulatory signals that control the expression of the *trp* biosynthetic genes. In particular, analyses of the mechanisms used to regulate expression of the *trp* genes in Gram-positive bacteria revealed the existence of two principal regulatory strategies. The first one, described in *Bacillus subtilis* and its closest relatives, involves a transcriptional attenuation mechanism mediated by the product of the gene *mtrB*, the Trp-activated RNA-binding protein TRAP [4, 5]. The second strategy, restricted to the Firmicutes, is based on the T-box mechanism. This enables a leader RNA to recognize, and respond to, uncharged tRNATrp, thus regulating *trp* gene expression [4, 6].

In *S*. *coelicolor* A3(2), tryptophan biosynthetic genes are organized in polycistronic operons (*trpCXBA* and *trpC2D2GE2*) and/or exist as single genes (*trpE3*, *trpE* and *priA*). Interestingly, *S*. *coelicolor* A3(2), differently from the actinobacterium *Corynebacterium*, lacks a *trpF* gene [7]. The lack of *trpF* is compensated by a dual-substrate phosphoribosyl isomerase, encoded by the *priA* gene, a close homolog of the *hisA* gene. Thus, the product of *priA* participates in the biosynthesis of both L-tryptophan and L-histidine [7, 8]. In addition, many of the seven *trp* genes have paralogs that map in separate operons, such as *trpC2D2GE2* genes within the gene cluster responsible for the production of calcium-dependent antibiotic (CDA), a cyclic lipopeptide antibiotic containing L- and D-Trp residues [9].

It is noteworthy that expression of the *trp* biosynthetic genes is not negatively controlled by Trp [10], which is different to what is observed in other bacteria [4]. In fact, *trp* gene expression is regulated in response to the general growth requirements of the cell [3] and their supplementation to minimal medium can stimulate the transcription of the *trp* genes, including those present in the gene cluster responsible for the biosynthesis of CDA [10].

The interconnection between metabolic pathways, the evolution of amino acid biosynthetic enzymes with promiscuous activities [11] and the presence of small ORFs in amino acid biosynthetic gene clusters is widely acknowledged in *S*. *coelicolor* A3(2) [12, 13]. Thus, other molecular mechanisms are proposed to exist in this actinomycete that may act at the post-transcriptional and/or translational levels. In order to investigate the role and function of the *SCO2038* smORF, a comprehensive study was performed in *S*. *coelicolor* A3(2) by combining bioinformatics, genetic manipulations, molecular and proteomic investigations and phenotypic analyses.

## Results and Discussion

### The *SCO2038* smORF is involved in tryptophan biosynthesis

**Fig 1:**
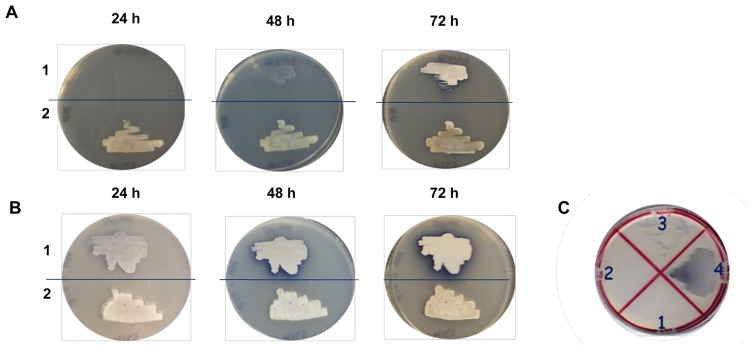
Growth of *S*. *coelicolor* 2038KO mutant strain on solid MM. Growth of 2038KO (1) and WT (2) on solid MM after 24, 48 and 72 h (A) and solid MM-Trp (B). (C) Growth of WT (2) (1), 2038KO (2), pKC796Hyg (3), pKC796Hyg-*sco2038* (4) on solid MM with the antibiotic hygromycin.

The *SCO2038* smORF located in *trpCXBA* locus putatively encodes an unknown protein highly conserved among streptomycetes, and which does not show similarity to any protein having a known function (see S1 File). In order to assess the involvement of the *SCO2038* product in Trp biosynthesis, we generated a *SCO2038* knockout mutant strain (hereafter called 2038KO) using the ReDirect PCR-targeting technology [14], where the coding region was replaced with an apramycin resistance (*apr*) cassette. Interestingly, we observed that the *SCO2038* deletion caused a 2 day-delay in growth of the 2038KO strain on minimal medium (MM), when compared to the wild type (WT) strain (Fig 1A). The phenotype of strain 2038KO was abolished by supplementation of Trp to the MM (MM-Trp) (Fig 1B) and by complementation of the mutation performed by introducing the SCO2038 gene into 2038KO using the pKC796Hyg vector (pKC796Hyg-SCO2038) (Fig 1C). The resulting complemented strain rescued the ability to grow on MM (Fig 1C). Moreover, this last result suggested that the *SCO2038* downstream genes *trpBA* present in *trpCXBA* locus can be expressed independently from *SCO2038*, as also confirmed by (q)RT-PCR (data not shown), thus excluding the possibility that the delayed growth was caused by a polar effect.

**Fig 2:**
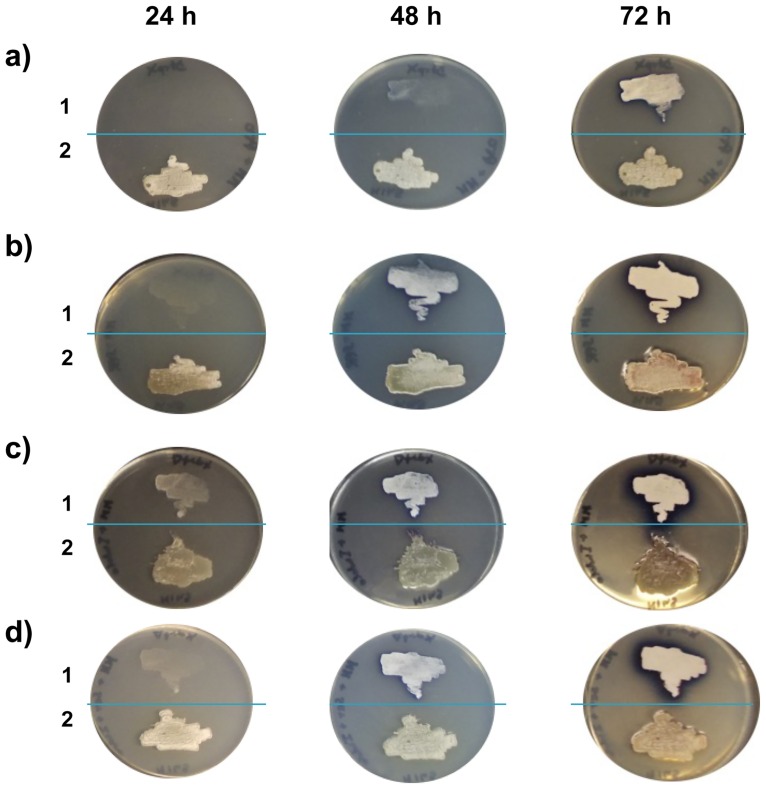
*S*. *coelicolor* 2038KO growth on MM supplemented with tryptophan precursors serine and indole. (a) Growth of 2038KO (1) and WT (2) on MM-Pro (negative control); b) MM-serine; c) MM-indole; d) MM-serine-indole.

All these results revealed that SCO2038 is involved in Trp biosynthesis. However, in contrast to the phenotype caused by mutations in trpB and trpA genes, which cause auxotrophy [15], the 2038KO mutant was characterized by a slow rate of growth on MM. This evidence suggests that the strain is still able to synthetize Trp but less efficiently than the WT strain. The enzymes encoded by the *trpCXBA* locus are involved in the last steps of Trp biosynthesis; in particular, TrpA produces indole from indole-3-glycerol phosphate supplied by TrpC, while TrpB condenses indole (Ind) and serine (Ser), which is obtained from the cellular pool, to form Trp. In order to assess whether the *SCO2038* gene product is involved in the last steps of the biosynthetic pathway leading to Trp production, we tested the effects of the supplementation of Trp precursors, namely Ser and Ind, on the growth of the mutant. Also in this case, both Ser and/or Ind specifically abolished the observed growth-delay, similarly to what was observed for Trp supplementation (Fig 2). Notably, addition of Ind worked better than addition of Ser. On the contrary, the supplementation of Ser and Ind to *trpA* and *trpB* knockout strains, previously obtained in our lab (unpublished data), did not abolish their inability of growing on MM (S1 Fig). Thus, these results indicated that SCO2038 is not involved in the Ser-Ind condensation reaction during the course of Trp biosynthesis. In addition, these results suggest that the cellular availability of Ser and Ind, which are diverted to Trp biosynthesis, is a bottleneck in the growth of the 2038KO mutant. Based on this evidence, together with the results described below, which revealed an involvement of SCO2038 in the modulation of Trp precursor biosynthesis, we therefore decided to change the original name *trpX* to *trpM* for Modulator of Trp biosynthesis; the corresponding *SCO2038* product is henceforth named TrpM.

### The *trpM* smORF affects morpho-physiological differentiation

**Fig 3:**
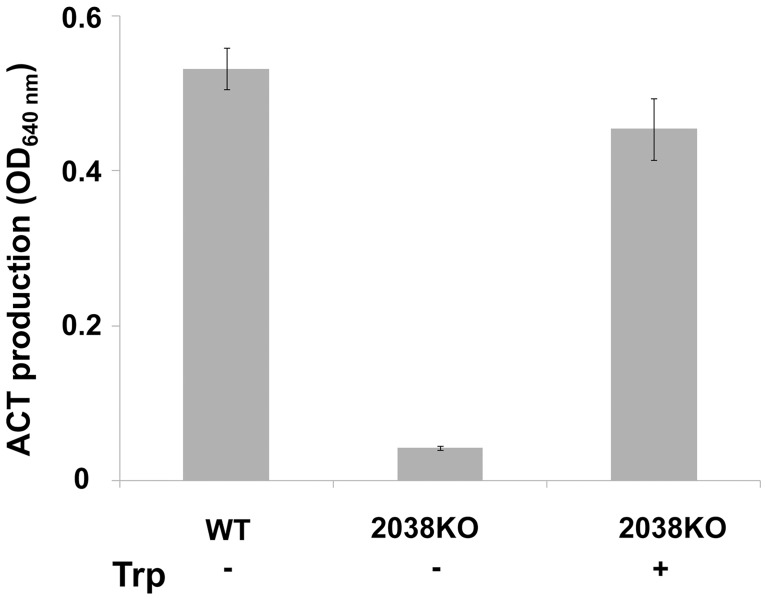
Actinorhodin antibiotic production of *S*. *coelicolor* 2038KO. Production of actinorhodin in 2038KO and WT on MM, and in 2038KO on MM-Trp after 4 days of incubation. Histograms report values from three different cultivations. Vertical bars represent standard deviations.

**Fig 4:**
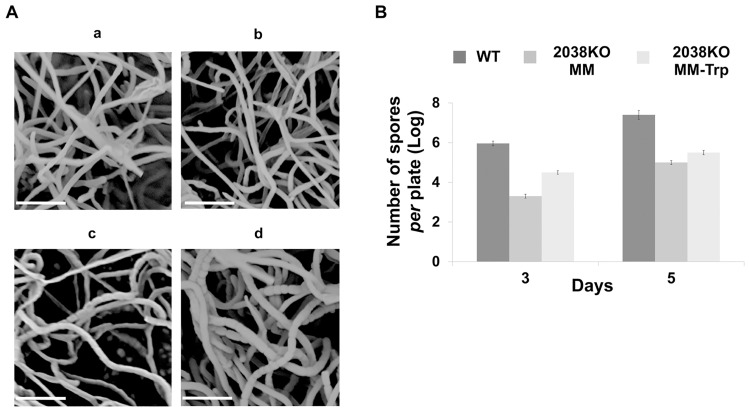
Role of *trpM* on morphological development. (A) SEM micrograph images of SCO2038KO after 5 days of growth on MM (a) and MM-Trp (b); WT (2) after 3 (c) and 5 (d) days of growth on MM; bars indicate 10 μm. (B) Quantification of spore production yield in 2038KO and WT strains after 3 and 5 days of growth on MM and MM-Trp. Histograms report values from three different cultivations. Vertical bars show standard deviations.

In *S*. *coelicolor* A3(2), Trp is used as an amino acid building block for protein biosynthesis and as carbon and nitrogen sources [10]. Consequently, it positively influences morphological differentiation and production of antibiotics by promoting the accumulation of: i) pleiotropic gene products associated with life-cycle progression; and ii) specific enzymes involved in many anabolic and catabolic process [10]. In order to evaluate the effect of the *SCO2038* deletion, production of the antibiotic actinorhodin (ACT) was quantitatively analyzed in the 2038KO strain using a spectrophotometric assay [16]. Results of this analysis revealed that the amount of ACT produced by the mutant strain was more than ten-fold lower than that produced by the WT (Fig 3). The addition of Trp to the 2038KO mutant cultures considerably increased ACT production (Fig 3), confirming the positive effect of Trp on ACT biosynthesis [10]. Furthermore, Scanning Electron Microscopy (SEM) analysis of strain 2038KO showed that morphological development was compromised. In particular, after 5 days of incubation on MM, the mutant strain showed small aerial hyphae, and immature spore chains with few and faint septal constrictions (Fig 4A). Trp supplementation modestly improved mycelium morphology and sporulation. This evidence was further corroborated by spore quantification (Fig 4B), which suggested the involvement of TrpM in the morphological differentiation of *S*. *coelicolor* A3(2).

### *S*. *coelicolor* A3(2) metabolic pathways affected by *trpM*

The effects that the *trpM* mutation exerted on growth rate, morphological development and antibiotic production in *S*. *coelicolor* A3(2) prompted us to investigate globally metabolic pathways and molecular processes specifically affected by the *trpM* deletion. To this end, a differential proteomic analysis based on 2D-difference *in gel* electrophoresis (2D-DIGE) and nano-liquid chromatography coupled to electrospray linear ion trap tandem mass spectrometry (nLC-ESI-LIT-MS/MS) was performed. In particular, a detailed comparative proteomic analysis was carried out on: i) the 2038KO strain grown on MM (2038KO MM); ii) the 2038KO strain grown on MM-Trp (2038KO MM-Trp); and iii) the WT strain grown on MM (WT MM) using 2038KO MM as pivotal condition (S2 Fig). Biomass samples were collected at the mid-exponential growth stages and the obtained proteome 2D-maps were comparatively evaluated. The protein species showing quantitative changes (*i*.*e*., fold change value of at least 1.5, with *p*<0.05) were identified by mass spectrometry procedures. The results of this analysis are shown in the S1 File. The abundance profiles of the identified protein species were categorized as constant (C, *i*.*e*., no significant quantitative changes with the respect to 2038KO MM), and either decreased (D) or increased (I). In order to highlight the influence of Trp addition over the loss of TrpM function, the differentially represented species were categorized into 8 possible groups, which emphasize concordant (II and DD), discordant (ID and DI) and heterogeneous (CI, CD, IC and DC) abundance profiles. In particular, the first and second positions in the acronyms refer to 2038KO MM *vs* WT MM and 2038KO MM *vs* 2038KO MM-Trp comparisons, respectively (S1 Table).

**Fig 5:**
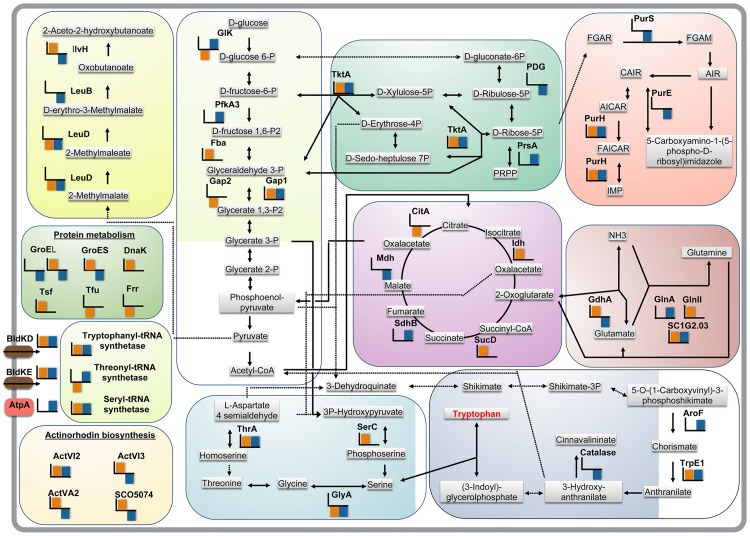
Global view of the metabolic pathways affected by *trpM* deletion. Metabolic pathways involving proteins over- and under-represented in 2038KO cultivated on MM *vs* WT cultivated on MM and in 2038KO cultivated on MM *vs* 2038KO cultivated on MM-Trp are shown with orange and blue bar color, respectively.

Indeed, quantitative changes in 2038KO MM *vs* WT MM and 2038KO MM *vs* 2038KO MM-Trp comparisons were evident for proteins involved in different molecular and metabolic processes, including amino acid biosynthesis, carbon and nitrogen metabolism, antibiotic production and morphological differentiation, in agreement with the described phenotypic observations. The role of these proteins is described in detail in the S1 File, with the exception of enzymes associated with Trp biosynthesis. In particular, proteins involved in the synthesis and utilization of Trp, or in the metabolism of Trp precursors and related intermediates were over-represented in the 2038KO MM with respect to WT. They are: i) anthranilate synthase (TrpE1), a paralogous gene conserved in *Streptomyces*, which catalyzes the conversion of chorismate into anthranilate [17]; ii) tryptophanyl-tRNA synthetase 1 (TrpS1); iii) phosphoserine aminotransferase (SerC) and homoserine dehydrogenase (ThrA), which are involved in the biosynthesis of Ser and threonine from central carbon metabolites, respectively (Fig 5). In addition, the under-representation of serine-hydroxymethyltransferase (GlyA), which is putatively devoted to the conversion of Ser to glycine (Gly) [18], suggests an increased availability of Ser in order to supply Trp biosynthesis (Fig 5). All these proteins had concordant abundance profiles in both 2038KO MM *vs* WT MM and 2038KO MM *vs* 2038KO MM-Trp comparisons, with the exception of SerC, whose IC profile suggested that its accumulation is due to the *trpM* mutation. Moreover, the transketolase (TktA), involved in the synthesis of D-erythrose-4-P, required for aromatic amino acid biosynthesis was over-represented in 2038KO MM. Interestingly, this was the only enzyme among those belonging to carbon metabolism group having an II profile, thus highlighting an increased need for this enzyme in the 2038KO MM condition. Similarly, the accumulation profiles of cysteine desulfurase (DD profile) and thiosulfate sulfurtransferase (CD profile) also suggested a relationship between sulphur metabolism and Trp synthesis/supplementation, probably due to the central role of Ser in both cysteine (Cys) and Trp biosynthesis, according to metabolic pathways of the KEGG2 database [19].

In conclusion, a *trpM* deletion was associated with an accumulation of proteins that are involved in biosynthetic steps of Trp precursors. Furthermore, proteome comparisons revealed that this accumulation might be dependent on either the cellular need for Trp or the loss of function of *trpM*, as in the case of SerC.

### TrpM-interacting protein partners

**Fig 6:**
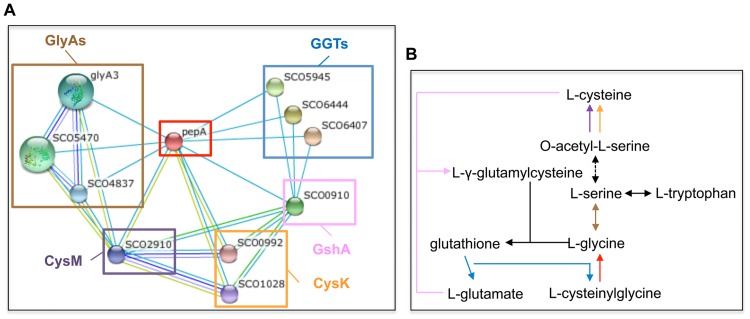
Protein SCO2179 (PepA) association network. (A) STRING interrogation showed PepA association with enzymes involved in amino acid metabolism: glycine hydroxymethyltransferase (GlyA); glutamate-cysteine ligase (GshA); cysteine synthases (CysK and CysM); glutathione hydrolase (GGT). (B) Representation of metabolic circuit involving PepA network partners.

A bioinformatic analysis of disordered/unstructured regions was performed on the TrpM amino acid sequence, using the DisEMBL computational tool [20]; it revealed that the N- and C-domains are disordered regions putatively involved in protein binding. In order to investigate the TrpM interactome, TrpM was overproduced as a His-tag protein in *E*. *coli* BL21(DE3)pLysS and purified by using metal-affinity chromatography (S3 Fig). Pull-down assay was performed using immobilized His-tagged TrpM as bait incubated with total protein extracts obtained from *S*. *coelicolor* A(3)2 grown on MM. SDS-PAGE and mass spectrometry identified ribosomal proteins S1 and S2, and putative leucyl aminopeptidase (PepA) in the prey protein fraction; they are the products of *SCO1998*, *SCO5624* and *SCO2179* genes, respectively. Interestingly, PepA was shown to play a role in *S*. *coelicolor* A3(2) antibiotic production and sporulation, although its molecular function has not yet been demonstrated [21]. Moreover, proteomic analysis carried out on a PepA disruption mutant strain showed an over-representation of GlyA, suggesting a negative control of PepA on GlyA [21]. The interaction between TrpM and PepA was confirmed by a bacterial adenylate cyclase two-hybrid assay (BACTH) experiment (S4 Fig) [22]. Interestingly, an *in silico* analysis by STRING database interrogation [23] suggested a PepA, GlyA, CysK and CysM network (Fig 6) involved in Ser, Gly and cysteine metabolism (Fig 6A). The central role of Ser was confirmed by the fact that the supplementation of Gly and Cys, as well as the addition of other amino acids such as tyrosine (Tyr), histidine (His) and proline (Pro), did not restore the growth rate of strain 2038KO. Thus, the interaction with PepA suggests that TrpM might control the intracellular pool of Ser via GlyA, consequently affecting the rate of Trp biosynthesis (Fig 6). These data are supported by proteomic analysis that showed a differential abundance of GlyA and of proteins involved in Cys and Trp metabolism in the 2038KO strain (Fig 5). Finally, a possible involvement of TrpM on translational control was suggested by the interaction with ribosomal proteins RpsA and RpsB. The latter, which was revealed as differentially represented in both 2038KO MM *vs* WT MM and 2038KO MM *vs* 2038KO MM-Trp proteomic comparisons (S1 Table), is involved in the formation of the translation initiation complex, where it contacts mRNA and allows ribonucleotide binding to 30S subunit RpsA, which in turn exerts a chaperone activity toward unfolded mRNAs [24, 25]. Further dedicated studies are necessary to confirm and to elucidate possible general or specific (possibly Trp dependent) roles for TrpM in controlling mRNA translation steps.

## Conclusions

This study provides novel insights on Trp biosynthesis in *S*. *coelicor*, identifying TrpM as a key component in the modulation of the Trp anabolic pathway and suggests a new model for the regulation of amino acid biosynthesis. Compared with the WT, strain 2038KO was characterized by delayed growth, smaller aerial hyphae and reduced production of spores and the antibiotic actinorhodin. Despite *S*. *coelicolor* A3(2) and closely related *Mycobacterium* species exhibiting a complex network connecting different amino acid biosynthetic pathways, such as those of Trp and His, through the evolution of phosphoribosyl isomerase A (PriA), which is a dual substrate enzyme [11, 12], the growth defect of strain 2038KO was only abolished by supplementation of Trp, and of its precursors Ser and Ind. The effect of the addition of Ind was slightly stronger than that of Ser. These findings suggest a preferential role of TrpM on Ind rather than on Ser biosynthesis, although the improved growth capability observed after indole addition may be consistent with possible stimulatory effects of this metabolite in additional cellular functions [26]. Thus, *trpM* gene retention in *Streptomyces* genome suggests that this small protein could be avoids metabolic conflicts related to substrate ambiguity in a dual substrate enzyme. In addition, proteomic results showed that TrpM exerts a negative control on the expression of TrpE1 and not on that of TrpE3, considered the central enzyme of the tryptophan biosynthetic pathway [4, 17]. Furthermore, the control of TrpM on TrpE1 could be explained by investigations on a double mutant strain.

Based to these phenotypic observations, proteomic analysis highlighted a role for TrpM in influencing the cellular pool of Trp precursors, Trp production and thus, bacterial growth and morpho-physiological differentiation. In fact, differential proteomics revealed a TrpM-dependent regulation of metabolic pathways and cellular processes that comprise enzymes involved in the biosynthesis of the Trp precursors Ser and Ind. Moreover, His-tag pull-down and BACTH assays, together with *in silico* analyses, corroborated the involvement of TrpM in a metabolic circuit involving Ser as key intermediate by mean of its interaction with PepA. Taken together, these data suggested a possible multi-tasking involvement of TrpM in various enzymatic reactions, thus highlighting a possible moon-lightning function. This characteristic is likely to be due to the versatility of disordered regions and loops within the protein, which allow different functional interactions [27, 28, 29]. Other proteins with moon-lightning activity have been predicted to occur in streptomycetes, such as the small proteins belonging to MbtH family. Despite the function of MbtH-like proteins still being unclear, they seem neverthless to play an important role in the production of many non-ribosomal peptide metabolites in bacteria, acting in mediating protein–protein interactions between components of NRPS biosynthetic systems important for metabolite assembly *in vivo*, post-transcriptional regulation, and metabolite export [2, 30]. This structural feature was also predicted for TrpM, due to the protein-interacting propensity of its N- and C-terminal domains. In conclusion, although further studies have to be carried out to elucidate the molecular mechanisms underlying how TrpM controls Trp biosynthesis, our data strongly suggest that TrpM plays a key role in a novel mechanism of metabolic regulation where amino acid biosynthesis is controlled by the modulation of precursor availability.

## Materials and Methods

### Bioinformatic analysis

A bioinformatic analysis of the *S*. *coelicolor* A3(2) genome was performed selecting small open reading frames encoding proteins with a molecular weight lower than 10 kDa and analyzing the chromosomal regions these genes. Neighboring genes with intergenic distances smaller or equal to 300 base pairs were considered to be associated with the analyzed small open reading frame. The amino acid sequence of the *SCO2038* gene product was used to perform BLAST interrogation against the National Center for Biotechnology Information (NCBI) database [31] and ClustalW sequence alignment [32].

### Bacteria strains, plasmid and growth conditions

*S*. *coelicolor* A(3)2, and *S*. *coelicolor* A(3)2 2038KO (S4 Table) were cultivated on soy flour-mannitol (SFM) agar [16] for spore suspension preparation and conjugal transfer. Solid minimal medium (MM) [16] and MM supplemented with 37 μg ml^-1^ Trp, 37 μg ml^-1^ Ser, 37 μg ml^-1^ Ind and 37 μg ml^-1^ Pro, 37 μg ml^-1^ Gly, 37 μg ml^-1^ Cys, 50 μg ml^-1^ His, 37 μg ml^-1^ Tyr were used for *S*. *coelicolor* A3(2) phenotypic analyses.

*E*. *coli* strains were grown at 37°C in Luria–Bertani (LB) medium supplemented with antibiotics as necessary (S5 Table).

### PCR and DNA manipulation

PCR reactions were performed under standard conditions. The identity of all DNA fragments amplified by PCR was confirmed by DNA sequencing. DNA manipulation, purification, ligation, restriction analysis, gel electrophoresis and transformation of *E*. *coli* were performed according to standard techniques [33].

Isolation of chromosomal DNA and transformation of *S*. *coelicolor* A3(2) were performed according to standard techniques [33]. The digoxigenin-11-dUTP labelling and detection kits (Roche Diagnostics GmbH, Mannheim, Germany) were used for the preparation of DNA probes and detection in Southern blot experiments, according to the protocols provided by the manufacturer.

### Construction of *SCO2038* deletion mutant and complementation

The strategy of PCR-targeting technology [14] was adopted for *S*. *coelicolor* A3(2) gene replacement to obtain the SCO2038KO mutant. In particular, two fragments flanking *SCO2038* were prepared by PCR with primer pairs described in S6 Table, and cloned on either side of an apramycin resistance (*apra*) cassette. The construct was then inserted into the vector pKC1132 [16], which was subsequently introduced into *S*. *coelicolor* A3(2) by conjugal transfer via ET12567/pUZ8002 [33, 34]. Transformants were then selected on MS medium containing apramycin. The *apra* cassette integration by double cross-over was confirmed by Southern blot and PCR analysis.

In order to generate a complemented pKC::*SCO2038* strains, the integrative plasmid pKC796Hyg [33] was digested with *Bam*HI and treated with alkaline phosphatase. The primers *SCO2038*comp Fw and *SCO2038*comp Rev (S6 Table) were used to amplify the fragments containing the *SCO2038* open reading frame and the upstream region, which contains the putative *sco2038* promoter, from the genomic DNA of *S*. *coelicolor* A3(2). The amplicon of *SCO2038* was first cloned into the pGEM-T vector (Promega). The amplicon was sequenced to confirm its identity, and then it was excised by *Bam*HI digestion and cloned into plasmid pKC796Hyg [33]. The resulting recombinant vector was used to transform *S*. *coelicolor* 2038KO mutant strain. The non-recombinant pKC796Hyg plasmid was used as a negative control in complementation assays. Correct plasmid integration into the *S*. *coelicolor* chromosome was verified by Southern hybridization, using as a probe the pKC796Hyg plasmid digested with *Pst*I and labelled by using digoxigenin-11-dUTP. The pKC796Hyg plasmid was a gift from Dr. Ramón I. Santamaría (Universidad de Salamanca, Spain).

### Measurement of actinorhodin production

To quantify total ACT production, 2038KO and *S*. *coelicolor* A3(2) cultures on MM and MM-Trp were stopped after 4 days of incubation and stored in 50-ml tubes (Corning, USA) at −20°C, overnight. Samples were then treated with 1 M KOH (final concentration), centrifuged at 3000 × *g* for 10 min, and the absorbance at 640 nm of the supernatant was measured [16].

### Spore production assay

Approximately 3x10^7^ spores of the respective strain were plated on soya mannitol (SM) agar in triplicate. The plates were incubated at 30°C for 3 and 5 days, respectively, before harvesting the newly formed spores. Serial dilutions of the spore samples were plated in duplicate to determine the spore titer.

### Electron microscopy observations

Electron microscopy observations were carried out using scanning electron microscopy (SEM) Phenom ProX, PhenomWorld, (The Netherlands) as previously described [10]. Spores and hyphae of the *S*. *coelicolor* A3(2) strain and *S*. *coelicolor* 2038KO mutant were observed after and 3 and 5 days of growth on agar MM or MM-Trp. Cut agar blocks were washed twice in phosphate-buffered saline (PBS) solution and then immersed in fixative solution (PBS, 2% (v/v) glutaraldehyde and 4% (v/v) paraformaldehyde) for 25 min, dehydratated and dried at 65°C. Each sample was sputter-coated with gold in order to avoid electrostatic charging under the electron beam and examined by SEM.

### Experimental set-up of differential proteome analysis

In order to perform biomass collection for differential proteome analysis between 2038KO MM, 2038KO MM-Trp and WT MM conditions growth kinetics were characterized by mean of biomass dry-weight measurements using four parallel replicas of each culture condition. In particular, 10^7^ spores of *S*. *coelicolor* A3(2) and *S*. *coelicolor* 2038KO strains were seeded on MM or MM supplemented with 37 μg ml^-1^ Trp and incubated at 30°C [16]. To facilitate mycelium harvesting cellophane disks placed directly onto the growth medium were used [10, 16]. Biomass samples, collected every 12 h, were dried (24 h at 65°C) and then weighed. Therefore, total proteins were extracted as previously described [35] from biomass samples collected from four parallel replicas of each condition during mid-exponential growth stages, which corresponded at 36–48 h for WT MM and 2038KO MM-Trp and 72–84 h for 2038KO MM.

### 2D-DIGE analysis

Protein extracts were labeled for 2D-DIGE analysis using CyDyeTM DIGE minimal labeling kit (GE Healthcare, Sweden), according to the manufacturer’s recommendations, including CyDye-swapping to avoid any labelling bias and using Cy2 dye to label the pooled protein internal standard [35]. Isoelectric focusing (IEF) was performed as previously described [35] on 3–10 pH range 18 cm-IPG strips (GE Healthcare, Sweden) using an Ettan IPGphor III apparatus (GE Healthcare, Sweden). After IEF, IPG strips were treated to separate focused proteins by using 12% SDS-PAGE in an Ettan Dalt six (GE Healthcare, Sweden) apparatus, with a maximum setting of 40 μA per gel and 110 V, at 10°C.

The 2D-gels images were digitalized using a DIGE imager (GE Healthcare) to detect cyanin-labeled proteins, and were analyzed using Image Master 2-D platinum version 7.0 (GE Healthcare, Sweden), as described previously [35]. Spots showing more than 1.5 fold change in spot volume (increased for over-representation or decreased for under-representation), with a statistically significant ANOVA value (*p*≤0.05), were considered as differentially represented and were further subjected to mass spectrometric analysis for protein identification. Samples for mass spectrometric identification were prepared by staining 2D-gels with ammoniacal silver [36], with minor modifications (0.08% w/v sodium thiosulfate for 5 min and 0.4% v/v silver solution for 30 min).

### Mass spectrometry analysis and protein identification

Protein spots and bands excised from 2D-gels and SDS-PAGE, respectively, were alkylated, digested with trypsin and identified [37]. Peptide mixtures were desalted by μgZip-TipC18 (Millipore, USA) using 50% v/v acetonitrile/5% v/v formic acid as eluent before nanoLC-ESI-LIT-MS/MS analysis. Tryptic digests were analyzed using a LTQ XL mass spectrometer (Thermo, USA) equipped with a Proxeon nanospray source connected to an Easy nanoLC (Thermo, USA). Peptide mixtures were separated on an Easy C18 column (10–0.075 mm, 3 μg) (Thermo, USA). Mobile phases were 0.1% v/v aqueous formic acid (solvent A) and 0.1% v/v formic acid in acetonitrile (solvent B), running at a total flow rate of 300 nL/min. A linear gradient was initiated 20 min after sample loading; solvent B ramped from 5% to 35% over 15 min, from 35% to 95% over 2 min. Spectra were acquired in the range *m/z* 400–1800. Acquisition was controlled by a data-dependent product ion scanning procedure over the 3 most abundant ions, enabling dynamic exclusion (repeat count 1 and exclusion duration 60 s); mass isolation window and collision energy were set to *m/z* 3 and 35%, respectively. MASCOT search engine version 2.2.06 (Matrix Science, UK) was used to identify protein spots from an updated NCBI non-redundant database (downloaded January 2015) also containing protein sequences for *S*. *coelicolor* A3(2), using nanoLC-ESI-LIT-MS/MS data. Database searching was performed selecting trypsin as proteolytic enzyme, a missed cleavages maximum value of 2, Cys carbamidomethylation and Met oxidation as fixed and variable modification, respectively. Candidates with at least 2 assigned peptides with an individual MASCOT score >25, both corresponding to *p*<0.05 for a significant identification, were further evaluated by the comparison with their calculated mass and p*I* values, using the experimental data obtained from electrophoresis.

### Overexpression and purification of TrpM-His_6_

For *trpM* overexpression, resulting in a TrpM-His_6_ protein, the entire ORF was amplified by PCR using the *S*. *coelicolor* A(3)2 genome as template and *SCO2028* Fw *Bam*HI and *SCO2038* Rev *Hind*III as primers (S6 Table). The amplicon was cloned into the pRSETB vector (Invitrogen) under the control of the IPTG-inducible promoter (S3 Table) and used to transform competent *E*. *coli* DH10B cells (Invitrogen). After selection of transformed *E*. *coli* cells and plasmid extraction, confirmation of *SCO2028* insertion into pRSETB was obtained by sequencing. Subsequently, the recombinant vector (pRSETB:: *sco2038*) was used to transform *E*. *coli* BL21(DE3)pLysS (Invitrogen) strain, where TrpM-His_6_ overexpression was conducted. In particular, the cells were grown at 37°C in LB medium supplemented with ampicillin and chloramphenicol (S5 Table) to 0.6 OD_600_ and then induced with IPTG (1 mM final concentration) for 3 h. After induction, the cells were harvested, suspended in 25 mM Tris-HCl (pH 7.1), 100 mM NaCl, 6 mM β-mercaptoethanol, 0.1 mM benzamidine and distrupted by sonication (output control 4, 4 × 15 s, Vibra Cell, USA). Cell debris and membrane fractions were separated from the soluble fraction by centrifugation (Sorvall SS34, 15,000 r.p.m., 45 min, 4°C). The supernatant containing the total protein extract was loaded on either Ni-NTA Agarose (Invitrogen) or Cobalt-containing Dynabeads (Invitrogen) and eluted following the manufacturer’s instructions. Eluted protein fractions were analyzed by 15% SDS-PAGE and TrpM-His_6_ over-expression was confirmed by Western-blot and mass spectrometric analyses.

### TrpM-His_6_ Pull-down assay

Pull-down assays were performed according to the manufacturer’s instruction (Invitrogen, 2009), with minor modifications. In order to immobilize overproduced His-tagged-TrpM, protein lysate (0.8 mg mL^-1^) from *E*. *coli* BL21(DE3)pLysS carrying pRSETB:: *sco2038* was incubated with 2 mg of cobalt-containing Dynabeads for 10 min. After five washing steps to remove non-specifically bound *E*. *coli* proteins, immobilized TrpM-His_6_ was used as bait to prey *S*. *coelicolor* A3(2) proteins extracted after 3 days of growth on MM. In order to remove *S*. *coelicolor* A3(2) proteins interacting non-specifically with Dynabeads alone, *S*. *coelicolor* A3(2) protein extract was pre-incubated with this resin for 10 min; then, the flow-through fraction was collected and used as prey proteins targets for TrpM-His_6_ in the pull-down assay. Captured proteins were then eluted according to the manufacturer’s recommendations, separated by 15% SDS-PAGE and protein bands were revealed by silver staining as described above. Parallel control experiments were performed to evaluate the non-specific protein pull-down using as bait 0.8 mg ml^-1^ of protein crude extract from *E*. *coli* BL21(DE3)pLysS containing the non-recombinant plasmid pRSETB.

SDS-PAGE patterns of captured proteins from the His-tagged-TrpM pull-down and control experiment were comparatively evaluated. Selected protein bands, present only in the electrophoretic patterns of His-tagged-TrpM pull-down, were excised, alkylated with iodoacetamide, digested with trypsin and analyzed by nanoLC-ESI-LIT-MS/MS as reported above. Corresponding areas in the electrophoretic patterns of control experiments were also processed to confirm the specificity of protein identification.

### Bacterial Adenylate Cyclase Two-Hybrid assay

The interaction *in vivo* between the TrpM and SCO2179 proteins was tested by the use of the bacterial adenylate cyclase two-hybrid system (BACTH) (Euromedex). The efficiency of complementation was quantified by measuring the corresponding cAMP levels or β-galactosidase activity [22]. Briefly, *SCO2038* and *SCO2179* genes were amplified by PCR reactions using *S*. *coelicolor* A3(2) chromosomal DNA as a template and primers described in S6 Table. The obtained amplicons were cloned into pKT25, PKNT25 and pUT18, pUT18C vectors in-frame with the T25 and T18 fragment open reading frames (S3 Table). Recombinant plasmids were checked by restriction enzyme digestion and subsequently by DNA sequencing. To test TrpM and SCO2179 interactions, different combinations of recombinant plasmid were used to co-transform competent *E*. *coli* DHM1 reporter cells (*cya*^*-*^). The interaction between TrpM and protein SCO2179 was tested by quantification of β-galactosidase activities in liquid cultures, according to the manufacturer’s recommendations, using o-nitrophenol-β-galactoside (ONPG) as a substrate (S5 Table).

## Supporting Information

S1 Fig Growth of *S*. *coelicolor trpA* (a) and *trpB* (b) knockout mutants on MM supplemented with Trp and Trp precursors serine and indole. (PDF)

S2 Fig 2D-proteome maps of whole protein extracts from 2038KO MM, 2038KO MM-Trp and WT MM. (PDF)

S3 Fig TrpM overexpression in *E*. *coli* BL21(DE3)pLysS and purification by Ni-NTA Agarose column. A) Coomasie-blue-stained 15% SDS–PAGE gel. Lane M, protein weight standard. Lane 1, total *BL21(DE3*)pLysS pRSETB::*SCO2038* lysate. Lane 2, purified His-tagged SCO2038 protein. B) Western blot analysis.
(PDF)

S4 Fig Bacterial adenylate cyclase two-hybrid assay (BACTH) experiments. The interaction of fusion proteins was tested as β-galactosidase activity A [U/mg] in *E*. *coli* cultures grown for 24 h. The assay showed a significant increase of the β-galactosidase activity for all the plasmid combinations tested, when compared to the negative control. TrpM (protein SCO2038) and protein SCO2179 were also tested for self-association ability.
(PDF)

S5 Fig Bioinformatic analysis of smORFs present in the *S*. *coelicolor* genome. (A) Relative distribution according to functional role of smORFs encoding proteins with a molecular mass less than 10 kDa. (B) Relative distribution according to the functional role of surrounding region (300 nt) genes of smORF encoding proteins with an unknown function.
(PDF)

S6 Fig *SCO2038* smORF is conserved among *Streptomycetes*. (A) ClustalW amino acid sequence alignment of TrpM homologues. (B) Schematic organization (not in scale) of the genetic regions surrounding *SCO2038* gene and some homologues.
(PDF)

S7 Fig Functional distribution of the differentially represented protein species. (A) Functional distribution of the differentially represented protein species in the whole extract from the proteomic comparison 2038KO MM *vs* WT MM. (B) Functional distribution of the differentially represented protein species in the whole extract from the proteomic comparison 2038KO MM *vs* 2038KO MM-Trp. The pie chart shows the distribution (in percentage) of the proteins into their functional classes. a) Amino acid metabolism; b) carbon metabolism; c) energy metabolism; d) metabolism of cofactors and vitamines; e) morphological-physiological differentiation; f) nucleotide metabolism; g) other; h) oxidoreduction; i) protein metabolism; j) unknown.
(PDF)

S8 Fig Quantitative distribution of differentially represented protein species according to their distribution in functional classes. Depiction of protein species over- (dark red bars) and under- (dark green bars) represented in the whole extracts of 2038KO cultivated on MM *vs* WT cultivated on MM; Depiction of protein species over- (light red bars) and under- (light green bars) represented in the whole extracts of 2038KO MM *vs* 2038KO cultivated on MM-Trp. a) Amino acid metabolism; b) carbon metabolism; c) energy metabolism; d) metabolism of cofactors and vitamines; e) morphological-physiological differentiation; f) nucleotide metabolism; g) other; h) oxidoreduction; i) protein metabolism; j) unknown.
(PDF)

S9 Fig Distribution of protein species into functional classes according to their abundance profile as resulted by combining 2038KO MM *vs* WT MM and 2038KO MM *vs* 2038KO MM-Trp proteomic comparisons. I, D and C stand for Increased, Decreased and Constant abundance profile, with the first and the second positions referring to the two different analyses, respectively. a) Amino acid metabolism; b) carbon metabolism; c) energy metabolism; d) metabolism of cofactors and vitamines; e) morphological-physiological differentiation; f) nucleotide metabolism; g) other; h) oxidoreduction; i) protein metabolism; j) unknown.
(PDF)

S1 File (DOCX)

S1 Table Description, functional classification, abundance profile and mass spectrometry identification parameters of differentially represented proteins identified from global proteomic analysis. (XLSX)

S2 Table Description, abundance profile and mass spectrometry identification parameters of differentially represented spots containing multiple protein components. (XLSX)

S3 Table List of plasmids used in this study. (XLSX)

S4 Table List of strains used in this study. (XLSX)

S5 Table List of antibiotics and concentration thereof used in this study. (XLSX)

S6 Table List of primers used in this study. (XLSX)
