# Supplementary material for: TrpM, a Small Protein Modulating Tryptophan Biosynthesis and Morpho-Physiological Differentiation in Streptomyces coelicolor A3(2)
Source: PLoS One. 2016 Sep 26;11(9):e0163422. doi: 10.1371/journal.pone.0163422 (PMC5036795; doi:10.1371/journal.pone.0163422)
Supplement: S1 Fig — (PDF) [file pone.0163422.s001.pdf]

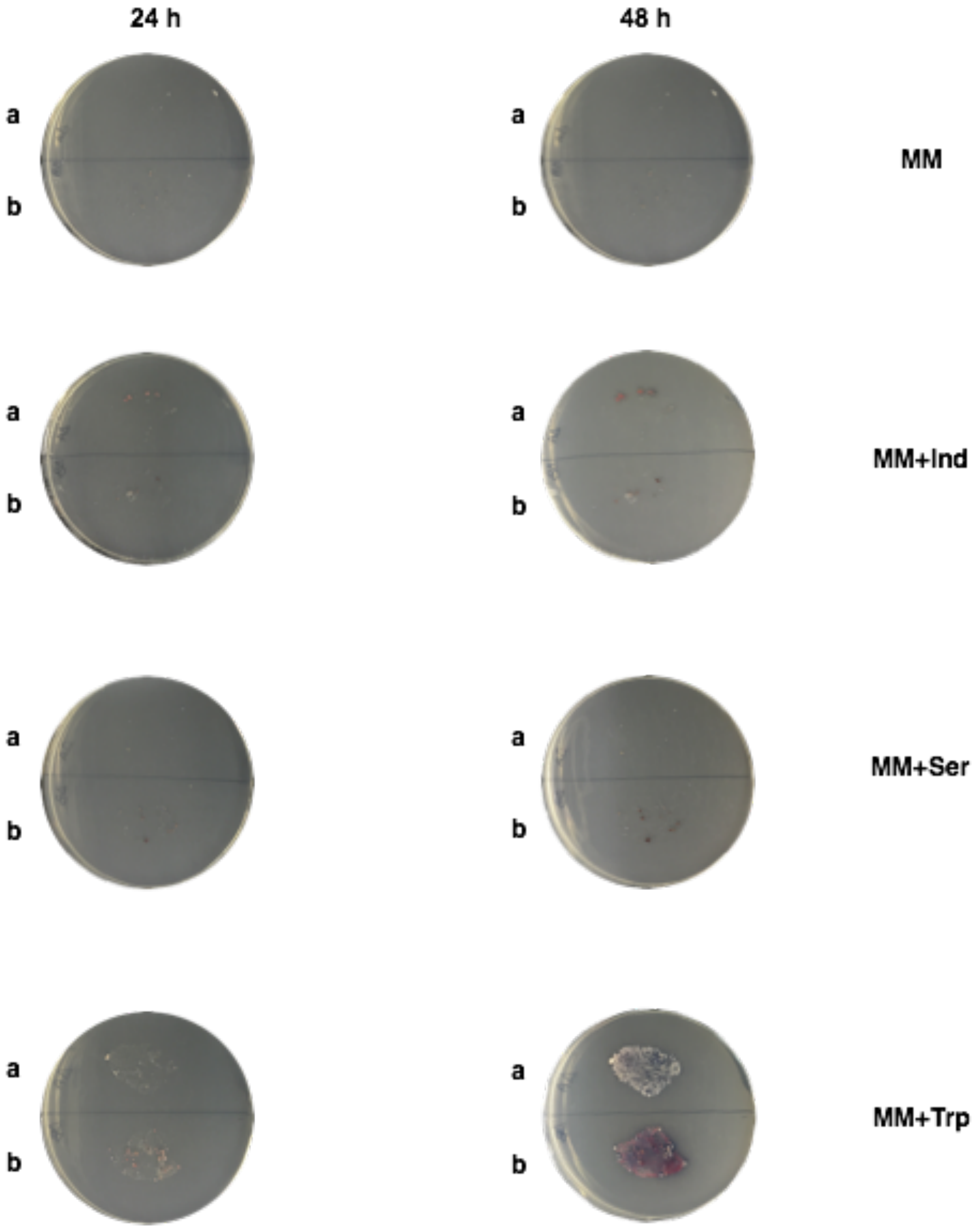

S1 Fig. Growth of *S. coelicolor trpA* (a) and *trpB* (b) knockout mutants on MM supplemented with Trp and Trp precursors serine and indole.
