# Supplementary figures and images for: TrpM, a Small Protein Modulating Tryptophan Biosynthesis and Morpho-Physiological Differentiation in Streptomyces coelicolor A3(2)
Source: PLoS One. 2016 Sep 26;11(9):e0163422. doi: 10.1371/journal.pone.0163422 (PMC5036795; doi:10.1371/journal.pone.0163422)

S2 Fig. 2D-proteome maps of whole protein extracts from 2038KO MM, 2038KO MM-Trp and WT MM.

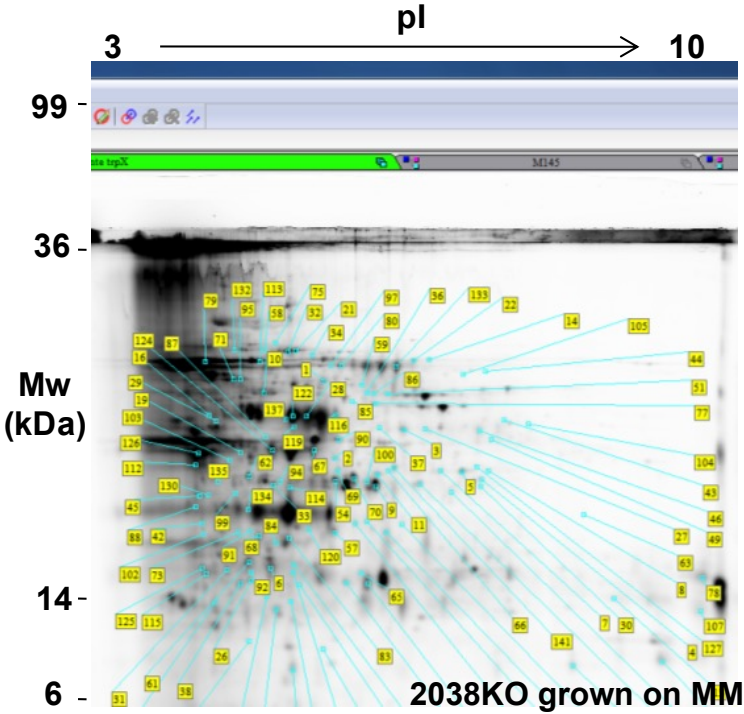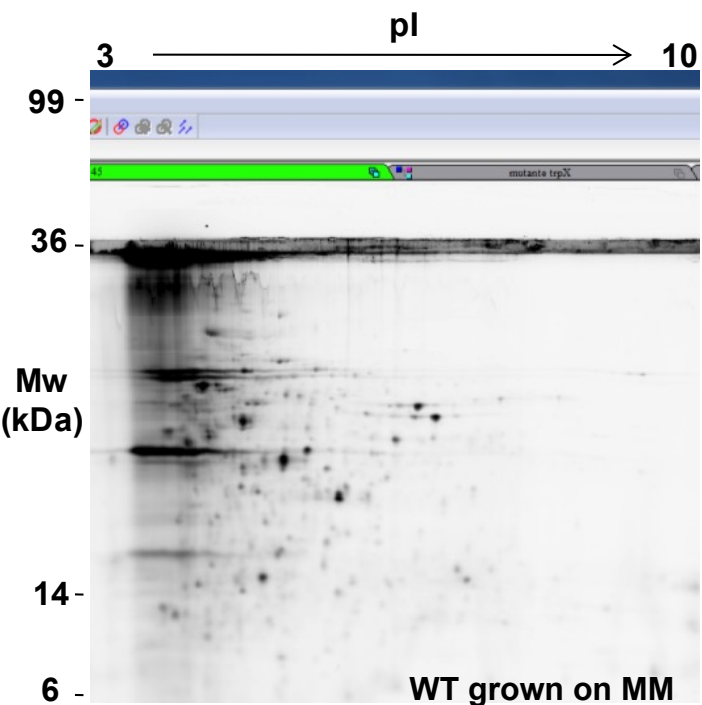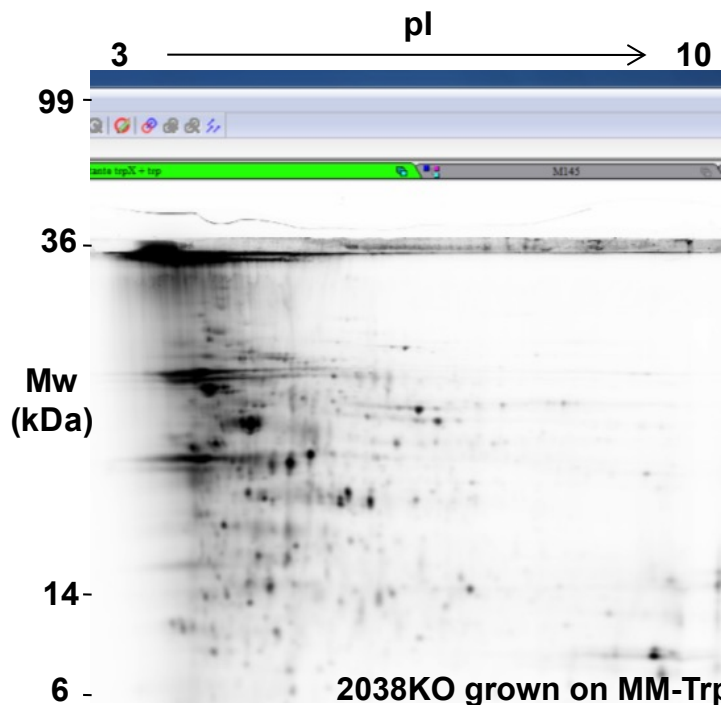

Supplement: S2 Fig — (PDF) [file pone.0163422.s002.pdf]

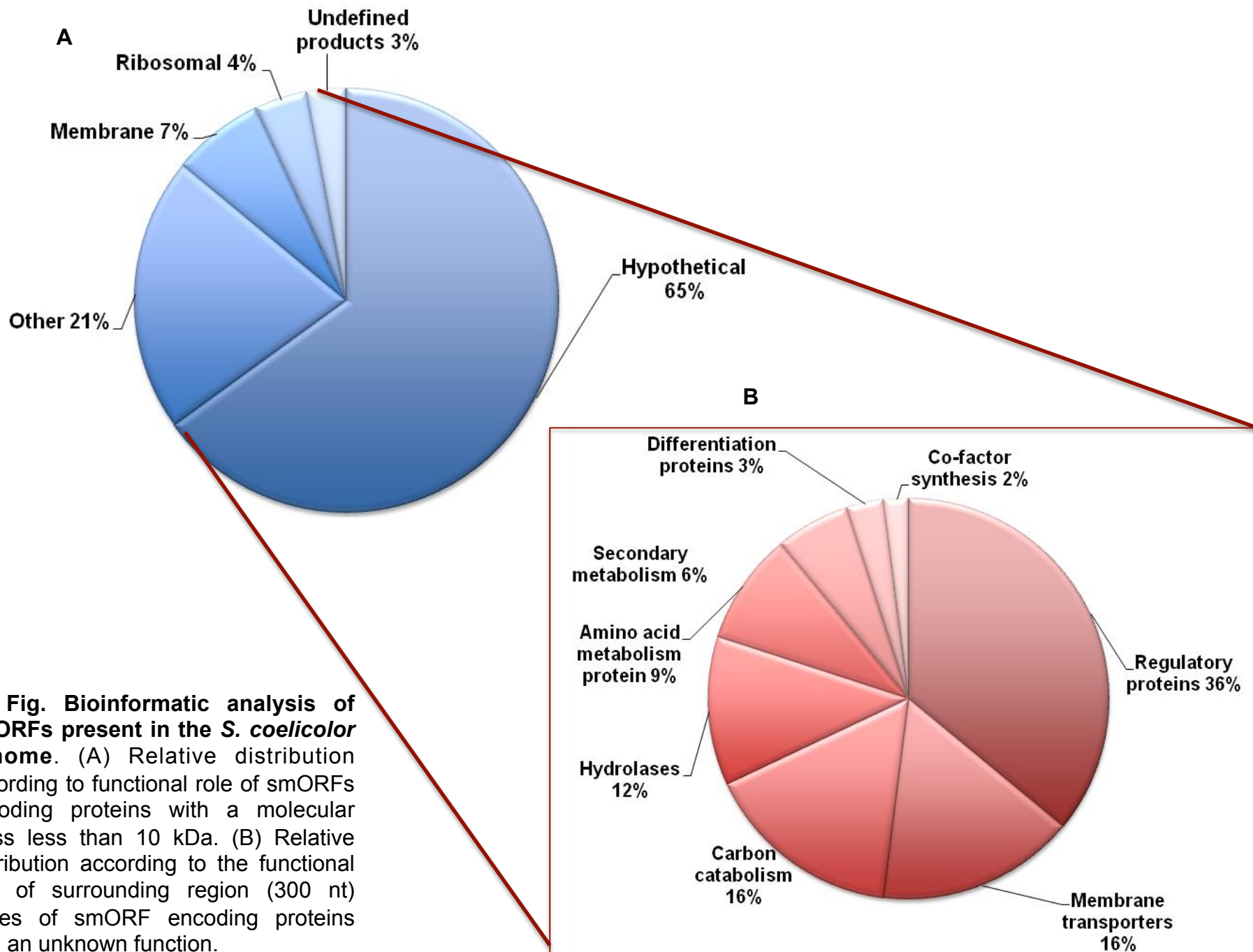

Supplement: S5 Fig — (A) Relative distribution according to functional role of smORFs encoding proteins with a molecular mass less than 10 kDa. (B) Relative distribution according to the functional role of surrounding region (300 nt) genes of smORF encoding proteins with an unknown function. (PDF) [file pone.0163422.s005.pdf]
