# Supplementary material for: TrpM, a Small Protein Modulating Tryptophan Biosynthesis and Morpho-Physiological Differentiation in Streptomyces coelicolor A3(2)
Source: PLoS One. 2016 Sep 26;11(9):e0163422. doi: 10.1371/journal.pone.0163422 (PMC5036795; doi:10.1371/journal.pone.0163422)
Supplement: S3 Fig — A) Coomasie-blue-stained 15% SDS–PAGE gel. Lane M, protein weight standard. Lane 1, total BL21(DE3)pLysS pRSETB::SCO2038 lysate. Lane 2, purified His-tagged SCO2038 protein. B) Western blot analysis. (PDF) [file pone.0163422.s003.pdf]

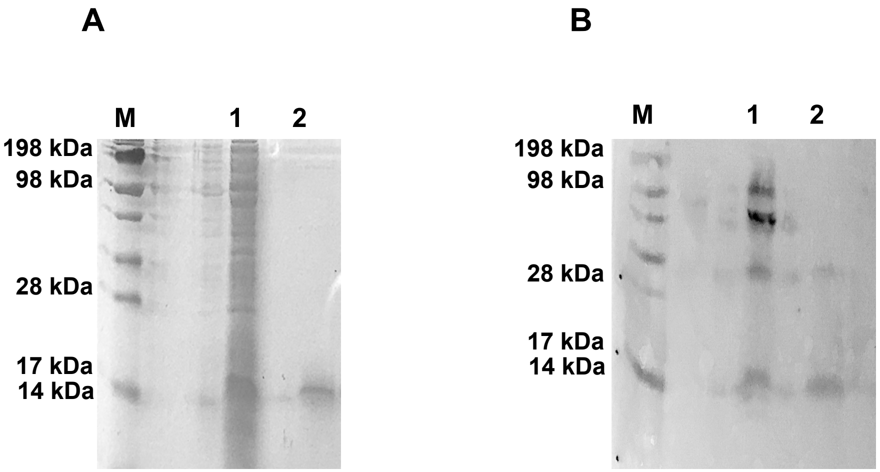

S3 Fig. SCO2038 overexpression in *E. coli* and purification by Ni-NTA Agarose column. A) Coomasie-blue-stained 15% SDS–PAGE gel. Lane M, protein weight standard. Lane 1, total *BL21(DE3*)pLysS pRSETB::*SCO2038* lysate. Lane 2, purified His-tagged SCO2038 protein. B) Western blot analysis.
