# Supplementary material for: TrpM, a Small Protein Modulating Tryptophan Biosynthesis and Morpho-Physiological Differentiation in Streptomyces coelicolor A3(2)
Source: PLoS One. 2016 Sep 26;11(9):e0163422. doi: 10.1371/journal.pone.0163422 (PMC5036795; doi:10.1371/journal.pone.0163422)
Supplement: S4 Fig — The interaction of fusion proteins was tested as β-galactosidase activity A [U/mg] in E. coli cultures grown for 24 h. The assay showed a significant increase of the β-galactosidase activity for all the plasmid combinations tested, when compared to the negative control. TrpM (protein SCO2038) and protein SCO2179 were also tested for self-association ability. (PDF) [file pone.0163422.s004.pdf]

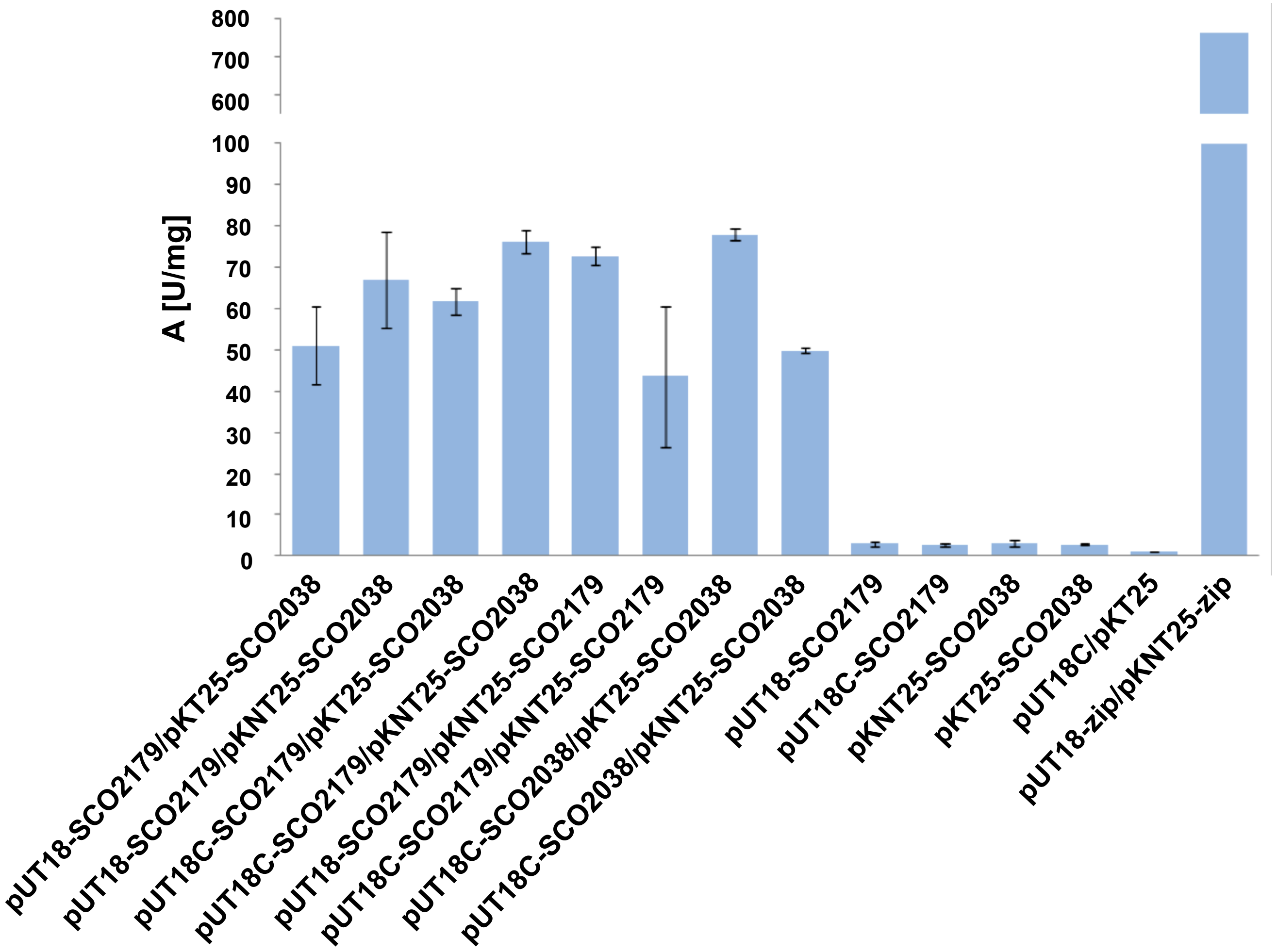

S4 Fig. Bacterial adenylate cyclase two-hybrid assay (BACTH) experiments. The interaction of fusion proteins was tested as β-galactosidase activity A [U/mg] in *E. coli* cultures grown for 24 h. The assay showed a significant increase of the β-galactosidase activity for all the plasmid combinations tested, when compared to the negative control. Proteins SCO2038 and SCO2179 were also tested for self-association ability.
