# Supplementary material for: TrpM, a Small Protein Modulating Tryptophan Biosynthesis and Morpho-Physiological Differentiation in Streptomyces coelicolor A3(2)
Source: PLoS One. 2016 Sep 26;11(9):e0163422. doi: 10.1371/journal.pone.0163422 (PMC5036795; doi:10.1371/journal.pone.0163422)
Supplement: S6 Fig — (A) ClustalW amino acid sequence alignment of TrpM homologues. (B) Schematic organization (not in scale) of the genetic regions surrounding SCO2038 gene and some homologues. (PDF) [file pone.0163422.s006.pdf]

A

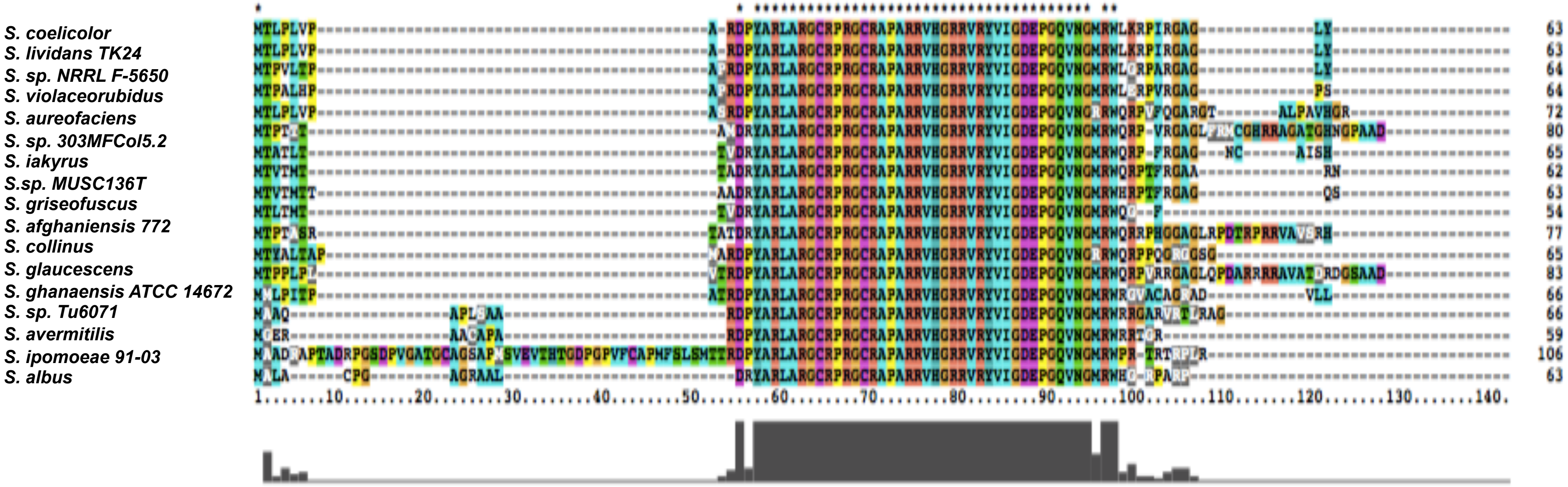

B

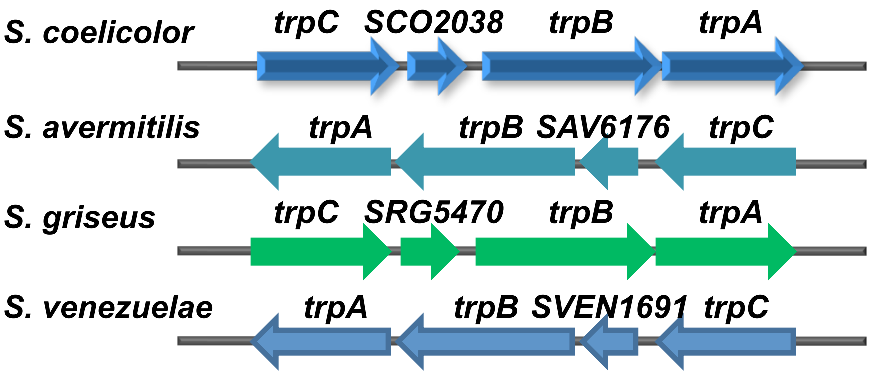

S6 Fig. smORF *SCO2038* is conserved among *Streptomycetes*. (A) ClustalW amino acid sequence alignment of protein SCO2038 homologues. (B) Schematic organization (not in scale) of the genetic regions surrounding *SCO2038* gene and some homologues.
