# Supplementary material for: TrpM, a Small Protein Modulating Tryptophan Biosynthesis and Morpho-Physiological Differentiation in Streptomyces coelicolor A3(2)
Source: PLoS One. 2016 Sep 26;11(9):e0163422. doi: 10.1371/journal.pone.0163422 (PMC5036795; doi:10.1371/journal.pone.0163422)
Supplement: S7 Fig — (A) Functional distribution of the differentially represented protein species in the whole extract from the proteomic comparison 2038KO MM vs WT MM. (B) Functional distribution of the differentially represented protein species in the whole extract from the proteomic comparison 2038KO MM vs 2038KO MM-Trp. The pie chart shows the distribution (in percentage) of the proteins into their functional classes. a) Amino acid metabolism; b) carbon metabolism; c) energy metabolism; d) metabolism of cofactors and vitamines; e) morphological-physiological differentiation; f) nucleotide metabolism; g) other; h) oxidoreduction; i) protein metabolism; j) unknown. (PDF) [file pone.0163422.s007.pdf]

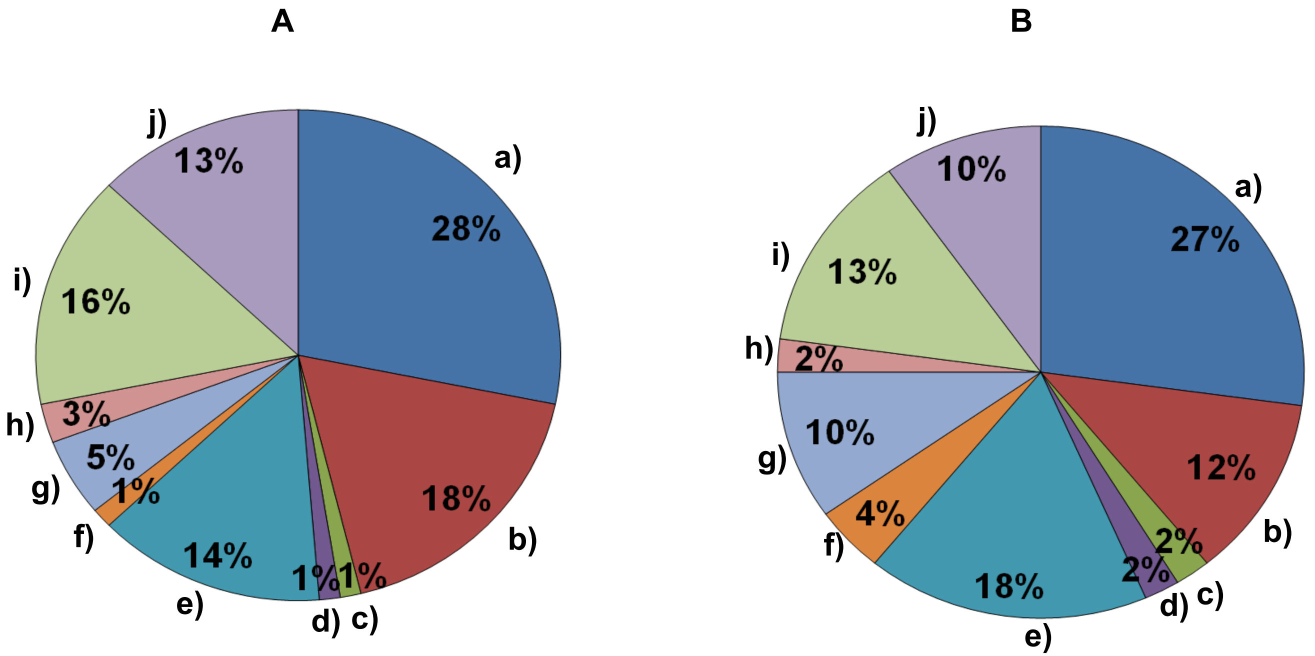

S7 Fig. Functional distribution of the differentially represented protein species. (A) Functional distribution of the differentially represented protein species in the whole extract from the proteomic comparison 2038KO *vs* WT both cultivated on MM. (B) Functional distribution of the differentially represented protein species in the whole extract from the proteomic comparison 2038KO cultivated on MM *vs* 2038KO cultivated on MM-Trp.
