# Supplementary material for: TrpM, a Small Protein Modulating Tryptophan Biosynthesis and Morpho-Physiological Differentiation in Streptomyces coelicolor A3(2)
Source: PLoS One. 2016 Sep 26;11(9):e0163422. doi: 10.1371/journal.pone.0163422 (PMC5036795; doi:10.1371/journal.pone.0163422)
Supplement: S8 Fig — Depiction of protein species over- (dark red bars) and under- (dark green bars) represented in the whole extracts of 2038KO cultivated on MM vs WT cultivated on MM; Depiction of protein species over- (light red bars) and under- (light green bars) represented in the whole extracts of 2038KO MM vs 2038KO cultivated on MM-Trp. a) Amino acid metabolism; b) carbon metabolism; c) energy metabolism; d) metabolism of cofactors and vitamines; e) morphological-physiological differentiation; f) nucleotide metabolism; g) other; h) oxidoreduction; i) protein metabolism; j) unknown. (PDF) [file pone.0163422.s008.pdf]

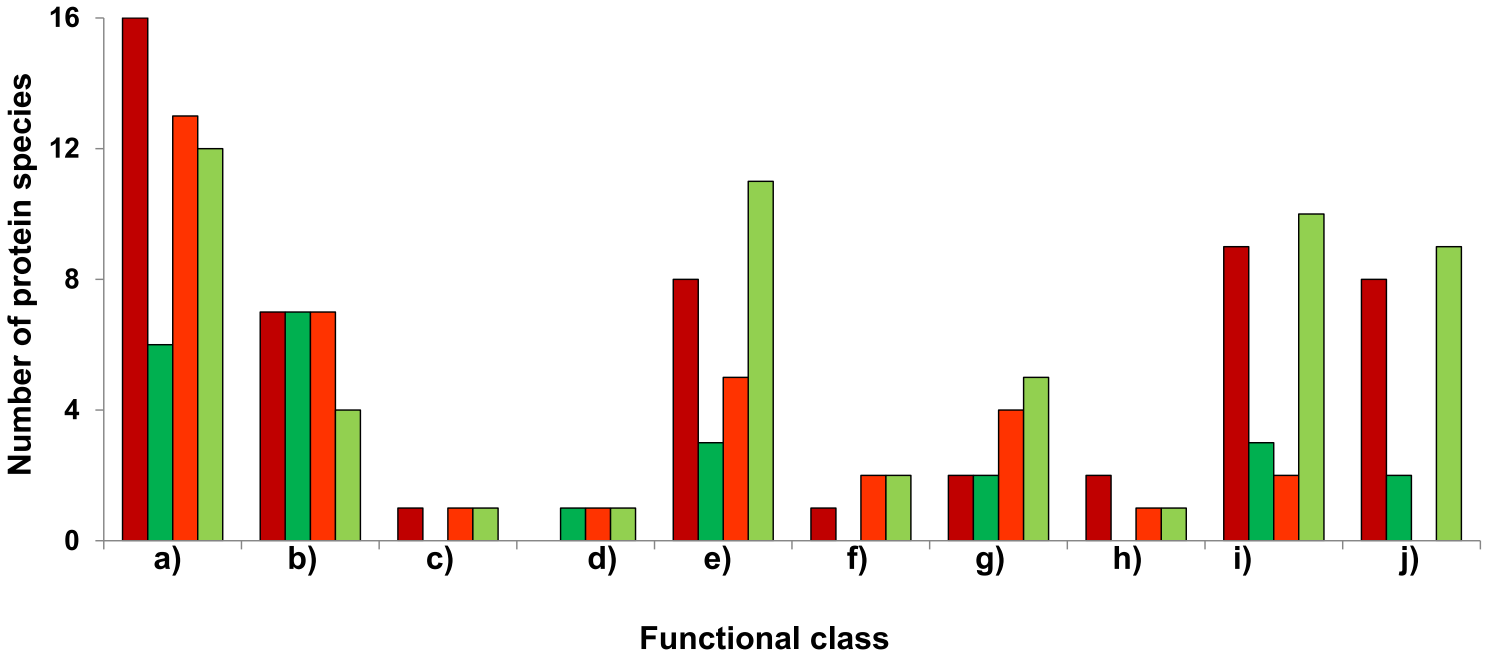

S8 Fig. Quantitative distribution of differentially represented protein species according to their distribution in functional classes. Depiction of protein species over- (dark red bars) and down- (dark green bars) represented in the whole extracts of 2038KO cultivated on MM vs WT cultivated on MM; Depiction of protein species over- (light red bars) and down- (light green bars) represented in the whole extracts of 2038KO cultivated on MM vs 2038KO cultivated on MM-Trp. a) Amino acid metabolism; b) carbon metabolism; c) energy metabolism; d) metabolism of cofactors and vitamines; e) morphological-physiological differentiation; f) nucleotide metabolism; g) other; h) oxidoreduction; i) protein metabolism; j) unknown.
