# Supplementary material for: TrpM, a Small Protein Modulating Tryptophan Biosynthesis and Morpho-Physiological Differentiation in Streptomyces coelicolor A3(2)
Source: PLoS One. 2016 Sep 26;11(9):e0163422. doi: 10.1371/journal.pone.0163422 (PMC5036795; doi:10.1371/journal.pone.0163422)
Supplement: S9 Fig — I, D and C stand for Increased, Decreased and Constant abundance profile, with the first and the second positions referring to the two different analyses, respectively. a) Amino acid metabolism; b) carbon metabolism; c) energy metabolism; d) metabolism of cofactors and vitamines; e) morphological-physiological differentiation; f) nucleotide metabolism; g) other; h) oxidoreduction; i) protein metabolism; j) unknown. (PDF) [file pone.0163422.s009.pdf]

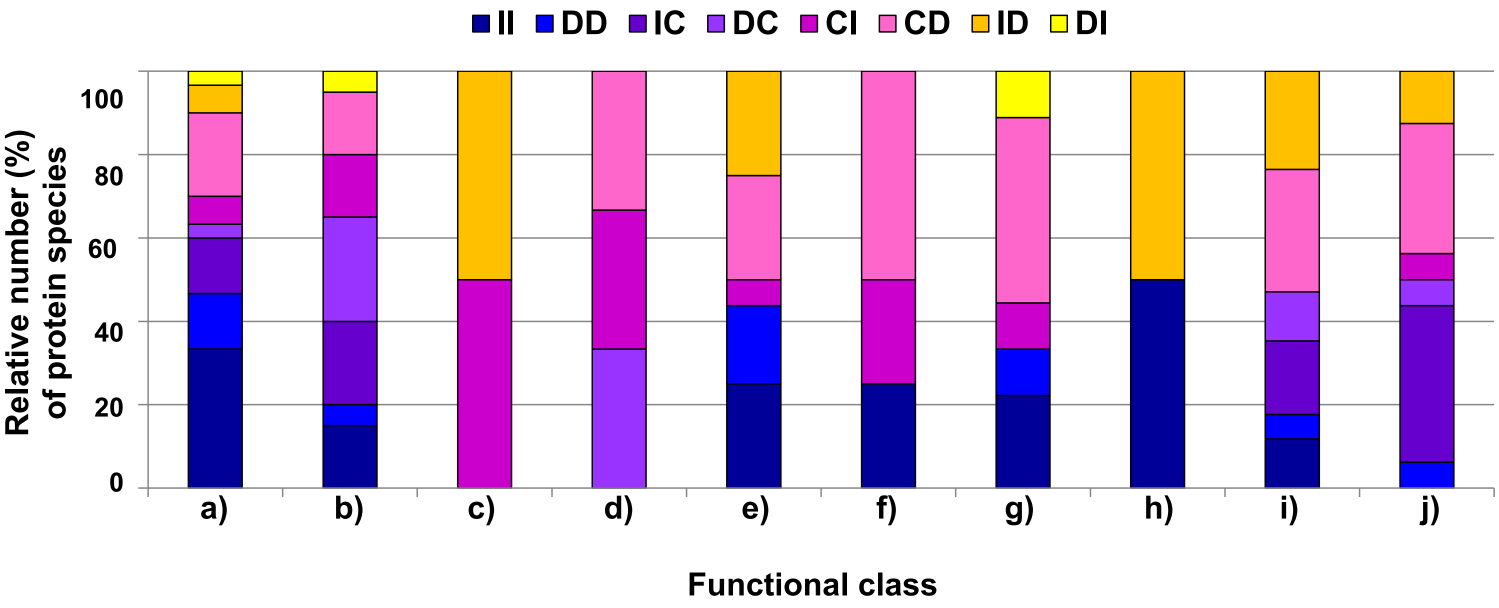

S9 Fig. Distribution of protein species into functional classes according to their abundance profile as resulted by combining "2038KO MM *vs* WT MM" and "2038KO MM *vs* 2038KO MM-Trp" proteomic comparisons. I, D and C stand for Increased, Decreased and Constant abundance profile, with the first and the second positions referring to the two different analyses, respectively. a) Amino acid metabolism; b) carbon metabolism; c) energy metabolism; d) metabolism of cofactors and vitamines; e) morphological-physiological differentiation; f) nucleotide metabolism; g) other; h) oxidoreduction; i) protein metabolism; j) unknown.
