# Supplementary material for: TrpM, a Small Protein Modulating Tryptophan Biosynthesis and Morpho-Physiological Differentiation in Streptomyces coelicolor A3(2)
Source: PLoS One. 2016 Sep 26;11(9):e0163422. doi: 10.1371/journal.pone.0163422 (PMC5036795; doi:10.1371/journal.pone.0163422)
Supplement: S1 File — (DOCX) [file pone.0163422.s010.docx]

**TrpM, a small protein modulating tryptophan biosynthesis and**

**morpho-physiological differentiation in *Streptomyces coelicolor* A3(2)**

Emilia Palazzotto*^,1,2^, Giuseppe Gallo^2^, Giovanni Renzone^3^, Anna Giardina^2^, Alberto Sutera^2^, Joohee Silva^2^, Celinè Vocat^2^, Luigi Botta^4^, Andrea Scaloni^3^, Anna Maria Puglia^2^

^1^Laboratory of Genetics, School of Biosciences and Veterinary Medicine, University of Camerino, 62032 Camerino, Italy;

^2^Laboratory of Molecular Microbiology and Biotechnology, STEBICEF Department, University of Palermo, 90128 Palermo, Italy

^3^Proteomic and Mass Spectrometry Laboratory, ISPAAM, National Research Council, 80147 Naples, Italy

^4^Dipartimento di Ingegneria Civile, Ambientale, Aerospaziale, dei Materiali, University of Palermo, 90128 Palermo, Italy

### Supporting information

## Abstract

In the model actinomycete *Streptomyces coelicolor* A3(2), small open reading frames encoding proteins with unknown functions were identified in several amino acid biosynthetic gene operons, such as *SCO2038* (*trpX*) in the tryptophan *trpCXBA* locus. In this study, the role of the corresponding protein in tryptophan biosynthesis was investigated by combining phenotypic and molecular analyses. The 2038KO mutant strain was characterized by delayed growth, smaller aerial hyphae and reduced production of spores and actinorhodin antibiotic, with respect to the WT strain.

The capability of this mutant to grow on minimal medium was rescued by tryptophan and tryptophan precursor (serine and/or indole) supplementation on minimal medium and by gene complementation, revealing the essential role of this protein, here named TrpM, as modulator of tryptophan biosynthesis. His-tag pull-down and bacterial adenylate cyclase-based two hybrid assays revealed TrpM interaction with a putative leucyl-aminopeptidase (PepA), highly conserved component among various *Streptomyces* spp.. In *silico* analyses showed that PepA is involved in the metabolism of serine, glycine and cysteine through a network including GlyA, CysK and CysM enzymes. Proteomic experiments suggested a TrpM-dependent regulation of metabolic pathways and cellular processes that includes enzymes such as GlyA, which is required for the biosynthesis of tryptophan precursors and key proteins participating in the morpho-physiological differentiation program. Altogether, these findings reveal that TrpM controls tryptophan biosynthesis at the level of direct precursor availability and, therefore, it is able to exert a crucial effect on the morpho-physiological differentiation program in *S. coelicolor* A3(2).

### Results

### SCO2038 protein is coded by a smORF conserved in streptomycetes

A bioinformatic analysis of the *S. coelicolor* genome allowed the identification of 502 smORFs, encoding proteins with a molecular mass lower than 10 kDa; they represent about 6.4% of the actinomycete genes. Among these genes, 68% are annotated as coding proteins with unknown function (UF), while 7% and 4% correspond to membrane and ribosomal component counterparts, respectively (S5 Fig). The analysis of the genomic regions surrounding the UF and membrane smORFs in the within of 300 bp showed that they are closed to ORFs encoding proteins involved in regulation, differentiation, secondary metabolism and biosynthesis of amino acids (S5 Fig). A prototype example is *SCO2038* (*trpX)* in the *trpCXBA* operon [1], which encodes a protein made of 63 amino acids, having a high content of Arg (22%), Gly (13%) and Pro (11%). This gene product has a an estimated *pI* value of 11.75 and a predicted mass of 7.167 Da. Bioinformatics revealed protein SCO2038 homologues to occur exclusively in streptomycetes, with a high amino acid sequence conservation (S6A Fig)*.* In addition, this analysis revealed that the organization of the *trpCXBA* *locus* is conserved among *Streptomyces* species (S6B Fig)*.*

**SCO2038-dependent proteomic changes**

The proteomic comparison 2038KO MM *vs* WT MM revealed 87 differentially represented protein species (*i.e.*, having a fold change value of at least 1.5, with *p*<0.05). Among them, 29 decreased and 58 increased their abundance in the 2038KO strain proteome (S1 and S2 Table). On the other hand, the 2038KO MM *vs* 2038KO MM-Trp proteomic comparison revealed 103 protein species showing quantitative changes (*i.e.*, fold change value of at least 1.5, with *p*<0.05), with 64 and 39 species having decreased and increased amounts in MM in the respect of MM-Trp, respectively (S1 and S2 Table). Since 58 protein spots were in common between the two data sets, a whole of 134 components was subjected to nLC-ESI-LIT-MS/MS analysis for protein identification (S1 and S2 Table). According to the putative functions, based on BLAST interrogations against KEGG2 database [2], the identified protein species were then clustered into 11 functional groups to obtain a global view of the metabolic and cellular processes modulated by *SCO2038* product expression (S7 and S8 Figs), or controlled by Trp supplementation in the genetic context of 2038KO (S7 and S8 Figs). Spots corresponding to multiple protein identification (15 in total) (S2 Table) were excluded from functional analysis. The most represented functional group in both 2038KO MM *vs* WT MM and 2038KO MM *vs* 2038KO MM-Trp proteomic comparisons was amino acid metabolism (29% and 28%, respectively; S7 Fig), whose members were mostly over-represented in 2038KO MM in both analyses (S8 Fig). Interestingly, about 47% of that showed a concordant abundance profile (*i.e*., II or DD), while only 10% of these proteins showed discordance trends (*i.e*., ID or DI) (S9 Fig).

Worth mentioning was also the differential representation of catalase (KatA) and 5-carboxymethyl-2-hydroxymuconate isomerase (HpaF), which are involved in the degradation pathway of aromatic compounds derived from Trp, and from Phe and Tyr, respectively [2, 3]. In particular, KatA showed a CD profile that is consistent with the external addition and accumulation of Trp in the actinomycete cultivation, while HpaF had an II profile, which can be associated with the catabolism and recycling of Phe and Tyr aromatic compound derivatives in a context of Trp lack.

Indeed, almost all (75%) proteins involved in carbon metabolism had an heterogeneous profile (*i.e*., CI, CD, IC and DC) in both analyses; this was associated with the fact that Trp may also be used as carbon and nitrogen source in *S. coelicolor*, as previously suggested [4], besides having the canonical role as amino acid building block for protein biosynthesis. This may hypothetically imply some effects on the abundance of central carbon and nitrogen metabolism proteins. In fact, the *SCO2038* mutation caused also the accumulation of enzymes required in glutamine/glutamate metabolism like glutamate dehydrogenase (GdhA, ID profile), which allows the interconversion of glutamate to the TCA cycle intermediate 2-oxoglutarate, and three glutamine synthetase paralogues (GlnA, CI profile; GlnII IC profile; SC1G2.03, II profile), catalyzing the conversion of glutamate into glutamine. These proteins control ammonia assimilation, together with the differentially abundant regulatory proteins GlnR and GlnK [5, 6], which showed CI and CD profiles, respectively. Taken together, these findings revealed a relationship between Trp metabolism, central carbon metabolism and ammonia assimilation, as previously reported [4].

A consistent number of key factors involved in actinomycete morpho-physiological differentiation were also differentially expressed in 2038KO MM *vs* WT MM and 2038KO MM *vs* 2038KO MM-Trp proteomic comparisons (13 and 18% of all differentially represented protein species, respectively; S7 Fig). Among them, BldKD and BldKE showed an II profile that is consistent with their role as members of the oligopeptide permease family of ABC transporters, which is encoded by the *bldKCDE* operon, whose function is associated with the early stages of morphological differentiation [7]. In fact, this transporter mediates the import of the morphogenic oligopeptide Bld261 thus inducing *S. coelicolor* differentiation [8, 9]. Moreover, proteins directly (*act* gene products) [10, 11] or indirectly involved in ACT production were found as differentially represented. In this context, worth mentioning are: i) the enzymes ActvA2 (SCO5077) and ActVA4 (SCO5079) showing the discordant profile ID; ii) two ActVI2 protein species showing an ID and II profile, respectively; iii) two species of the oligopeptide-binding lipoprotein having both a CD profile and whose gene *locus* (SCO5476-SCO5480) is associated with the stimulation of ACT production [12, 13]. Their expression profile revealed a stimulatory effect of Trp supplementation on *act* gene product, according to previous observations [4]. However, the over-representation in 2038KO MM of *act* on gene products in the respect of WT MM condition was not sufficient to ensure high levels of ACT production. A possible explanation of this phenomenon may be related to two aspects of antibiotic production in actinomycetes: i) the expression of genes involved in secondary metabolism is exerted as a consequence of stressing conditions/factors [14, 15]; ii) the supply of precursors from primary metabolism (in this case acetyl-CoA from glycolysis) is necessary to ensure good levels of antibiotic production [16, 17]. Accordingly, the slow growth rate observed for 2038KO strain and the corresponding Trp lack, which diverts primary metabolism intermediates towards amino acid biosynthesis, may stimulate *act* gene expression (Fig 5). In line with a stressed condition of 2038KO we observed the over-representation of the stress-induced proteins GroEL, GroES and DnaK chaperones [14] in 2038KO.

**TrpM-PepA interaction by BATCH assay**

To confirm the results of the pull-down assay and in order to examine *in vivo* the physical interaction between *SCO2038* and *SCO2179* expression products, we set out a BACTH experiment based on functional complementation between *Bordetella pertussis* adenylate cyclase fragments, namely T18 and T25, which are expressed separately from two compatible plasmids [18]. *SCO2038* and *SCO2179* sequences were cloned in frame with T25 and T18 genes in pKT25, pKNT25, pUT18 and pUT18C, as reported in S3 Table. The interaction of fusion proteins was tested pairwise by their ability to reconstitute active adenylate cyclase in the *E. coli* test strain DHM1, and was quantified by measuring the levels of β-galactosidase activity in liquid cultures. In *E. coli* DHM1 co-trasformed with all combinations of recombinant plasmid pairs, the assay showed a significant increase of the β-galactosidase activity for all the combinations, when compared to the negative control (S4 Fig). Moreover, *SCO2038* and *SCO2179* expression products were tested for self-association ability (S4 Fig; S3 Table), revealing that the two proteins can form homodimers or homomultimers, as already demonstrated for PepA in *E. coli* [19].

**Figure legend**

**S1 Fig. Growth of *S. coelicolor* *trpA* (a) and *trpB* (b) knockout mutants on MM supplemented with Trp and Trp precursors serine and indole.**

**S2 Fig. 2D-proteome maps of whole protein extracts from 2038KO MM, 2038KO MM-Trp and WT MM**.

**S3 Fig. TrpM overexpression in *E. coli*** BL21(DE3)pLysS **and purification by Ni-NTA Agarose column.** A) Coomasie-blue-stained 15% SDS–PAGE gel. Lane M, protein weight standard. Lane 1, total *BL21(DE3*)pLysS pRSETB::*SCO2038* lysate. Lane 2, purified His-tagged SCO2038 protein. B) Western blot analysis.

**S4 Fig. Bacterial adenylate cyclase two-hybrid assay (BACTH) experiments.** The interaction of fusion proteins was tested as β-galactosidase activity A [U/mg] in *E. coli* cultures grown for 24 h. The assay showed a significant increase of the β-galactosidase activity for all the plasmid combinations tested, when compared to the negative control. TrpM (protein SCO2038) and protein SCO2179 were also tested for self-association ability.

**S5 Fig. Bioinformatic analysis of smORFs present in the *S. coelicolor* genome**. (A) Relative distribution according to functional role of smORFs encoding proteins with a molecular mass less than 10 kDa. (B) Relative distribution according to the functional role of surrounding region (300 nt) genes of smORF encoding proteins with an unknown function.

**S6 Fig. *SCO2038* smORF is conserved among *Streptomycetes.*** (A) ClustalW amino acid sequence alignment of TrpM homologues. (B) Schematic organization (not in scale) of the genetic regions surrounding *SCO2038* gene and some homologues.

**S7 Fig. Functional distribution of the differentially represented protein species**. (A) Functional distribution of the differentially represented protein species in the whole extract from the proteomic comparison 2038KO MM *vs* WT MM. (B) Functional distribution of the differentially represented protein species in the whole extract from the proteomic comparison 2038KO MM *vs* 2038KO MM-Trp. The pie chart shows the distribution (in percentage) of the proteins into their functional classes. a) Amino acid metabolism; b) carbon metabolism; c) energy metabolism; d) metabolism of cofactors and vitamines; e) morphological-physiological differentiation; f) nucleotide metabolism; g) other; h) oxidoreduction; i) protein metabolism; j) unknown.

**S8 Fig.** **Quantitative distribution of differentially represented protein species according to their distribution in functional classes.** Depiction of protein species over- (dark red bars) and under- (dark green bars) represented in the whole extracts of 2038KO cultivated on MM *vs* WT cultivated on MM; Depiction of protein species over- (light red bars) and under- (light green bars) represented in the whole extracts of 2038KO MM *vs* 2038KO cultivated on MM-Trp. a) Amino acid metabolism; b) carbon metabolism; c) energy metabolism; d) metabolism of cofactors and vitamines; e) morphological-physiological differentiation; f) nucleotide metabolism; g) other; h) oxidoreduction; i) protein metabolism; j) unknown.

**S9 Fig. Distribution of protein species into functional classes according to their abundance profile as resulted by combining 2038KO MM *vs* WT MM and 2038KO MM *vs* 2038KO MM-Trp proteomic comparisons.** I, D and C stand for Increased, Decreased and Constant abundance profile, with the first and the second positions referring to the two different analyses, respectively. a) Amino acid metabolism; b) carbon metabolism; c) energy metabolism; d) metabolism of cofactors and vitamines; e) morphological-physiological differentiation; f) nucleotide metabolism; g) other; h) oxidoreduction; i) protein metabolism; j) unknown.

**Table legend**

**S1 Table.** **Description, functional classification, abundance profile and mass spectrometry identification parameters of differentially represented proteins identified from global proteomic analysis.**

**S2 Table.** **Description, abundance profile and mass spectrometry identification parameters of differentially represented spots containing multiple protein components.**

**S3 Table. List of plasmids used in this study.**

**S4 Table. List of strains used in this study.**

**S5 Table. List of antibiotics and concentration thereof used in this study.**

**S6 Table. List of primers used in this study.**

**References**

1. Hu DS, Hood DW, Heidstra R, Hodgson DA. The expression of the *trpD*, *trpC* and *trpBA* genes of *Streptomyces coelicolor* A3(2) is regulated by growth rate and growth phase but not by feedback repression. Mol Microbiol. 1999; 32: 869-80.
2. Kanehisa M, Goto S. KEGG: kyoto encyclopedia of genes and genomes. Nucleic Acids Res. 2000; 28: 27-30.
3. Chelikani P, Fita I, Loewen PC. Diversity of structures and properties among catalases. Cell Mol Life Sci. 2004; 61:192–208.
4. Palazzotto E, Renzone G, Fontana P*,* Botta, L, Scaloni A, Puglia AM, et al. Tryptophan promotes morphological and physiological differentiation in *Streptomyces coelicolor*. Appl Microbiol Biotechnol. 2015; 99: 10177-89.
5. [Fink D](http://www.ncbi.nlm.nih.gov/pubmed/?term=Fink%20D%5BAuthor%5D&cauthor=true&cauthor_uid=12406212), [Weissschuh N](http://www.ncbi.nlm.nih.gov/pubmed/?term=Weissschuh%20N%5BAuthor%5D&cauthor=true&cauthor_uid=12406212), [Reuther J](http://www.ncbi.nlm.nih.gov/pubmed/?term=Reuther%20J%5BAuthor%5D&cauthor=true&cauthor_uid=12406212), [Wohlleben W](http://www.ncbi.nlm.nih.gov/pubmed/?term=Wohlleben%20W%5BAuthor%5D&cauthor=true&cauthor_uid=12406212), [Engels A](http://www.ncbi.nlm.nih.gov/pubmed/?term=Engels%20A%5BAuthor%5D&cauthor=true&cauthor_uid=12406212). Two transcriptional regulators GlnR and GlnRII are involved in regulation of nitrogen metabolism in *Streptomyces coelicolor* A3(2). Mol Microbiol. 2002;46:331-47.
6. Yao LL, Liao CH, Huang G, Zhou Y, Rigali S, Zhang B, et al*.* GlnR-mediated regulation of nitrogen metabolism in the actinomycete *Saccharopolyspora erythraea*. Appl Microbiol Biotechnol. 2014; 98: 7935-48.
7. Kim DW, Chater KF, Lee KJ, Hesketh A. Effects of growth phase and the developmentally significant *bldA*-specified tRNA on the membrane-associated proteome of *Streptomyces coelicolor*. Microbiology*.* 2005; 151: 2707-20.
8. Nodwell JR, McGovern K, Losick R. An oligopeptide permease responsible for the import of an extracellular signal governing aerial mycelium formation in *Streptomyces coelicolor*. Mol Microbiol. 1996; 22: 881-93.
9. Akanuma G, Ueki M, Ishizuka M, Ohnishi Y, Horinouchi S*.* Control of aerial mycelium formation by the BldK oligopeptide ABC transporter in *Streptomyces griseus*. FEMS Microbiol Lett. 2011; 315: 54-62.
10. Okamoto S, Taguchi T, Ochi K, Ichinose K. Biosynthesis of actinorhodin and related antibiotics: discovery of alternative routes for quinone formation encoded in the act gene cluster. Chem Biol. 2009;16:226-36. doi: 10.1016/j.chembiol.2009.01.015.
11. Taguchi T, Itou K, Ebizuka Y, Malpartida F, Hopwood DA, Surti CM, et al. Chemical characterisation of disruptants of the *Streptomyces coelicolor* A3(2) *actVI* genes involved in actinorhodin biosynthesis. J Antibiot (Tokyo). 2000;53:144-52.
12. Meng L, Yang SH, Palaniyandi SA, Lee SK, Lee IA, Kim TJ, et al. Phosphoprotein affinity purification identifies proteins involved in S-adenosyl-L-methionine-induced enhancement of antibiotic production in *Streptomyces coelicolor*. J Antibiot (Tokyo). 2011; 64: 97-101.
13. Shin SK, Park HS, Kwon HJ*,* Yoon HJ, Suh JW. Genetic characterization of two S-adenosylmethionine-induced ABC transporters reveals their roles in modulations of secondary metabolism and sporulation in *Streptomyces coelicolor* M145. J Microbiol Biotechnol. 2007; 17: 1818-25.
14. Yang YH, Song E, Kim EJ, Lee K, Kim WS, Park SS, et al. NdgR, an IclR-like regulator involved in amino-acid-dependent growth, quorum sensing, and antibiotic production in *Streptomyces coelicolor*. Appl Microbiol Biotechnol. 2009;82:501-11. doi: 10.1007/s00253-008-1802-x. Epub 2008 Dec 13.
15. Godinez O, Dyson P, del Sol R*,* Barrios-Gonzalez J, Millan-Pacheco C, Mejia A. Targeting the osmotic stress response for strain improvement of an industrial producer of secondary metabolites. J Microbiol Biotechnol. 2015; 25: 1787-95
16. Butler MJ, Bruheim P, Jovetic S, Marinelli F, Postma PW, Bibb MJ*.* Engineering of primary carbon metabolism for improved antibiotic production in *Streptomyces lividans*. Appl Environ Microbiol. 2002; 68: 4731-9.
17. Olano C, Lombò F, Mendez C, Salas JA. Improving production of bioactive secondary metabolites in actinomycetes by metabolic engineering. Metab Eng. 2008; 10: 281-92.
18. Karimova G, Pidoux J, Ullmann A, Ladant D. A bacterial two-hybrid system based on a reconstituted signal transduction pathway. Proc Natl Acad Sci USA. 1998; 95: 5752-6.
19. Sträter N, Sherratt DJ, Colloms SD. X-ray structure of aminopeptidase A from *Escherichia coli* and a model for the nucleoprotein complex in Xer site-specific recombination. EMBO J. 1999;18:4513-22.
